# A Structural Analysis of the Angucycline-Like Antibiotic Auricin from *Streptomyces lavendulae* Subsp. *Lavendulae* CCM 3239 Revealed Its High Similarity to Griseusins

**DOI:** 10.3390/antibiotics8030102

**Published:** 2019-07-25

**Authors:** Maria Matulova, Lubomira Feckova, Renata Novakova, Erik Mingyar, Dominika Csolleiova, Martina Zduriencikova, Jan Sedlak, Vladimir Patoprsty, Vlasta Sasinkova, Iveta Uhliarikova, Beatrica Sevcikova, Bronislava Rezuchova, Dagmar Homerova, Jan Kormanec

**Affiliations:** 1 Institute of Chemistry, Slovak Academy of Sciences, 845 38 Bratislava, Slovakia; 2 Institute of Molecular Biology, Slovak Academy of Sciences, 845 51 Bratislava, Slovakia; 3 Cancer Research Institute BMC, Slovak Academy of Sciences, 845 05 Bratislava, Slovakia

**Keywords:** antibiotic, angucycline, auricin, biosynthesis, griseusin, polyketide, pyronaphthoquionone, regulation, secondary metabolism, *Streptomyces*, structure elucidation

## Abstract

We previously identified the *aur1* gene cluster in *Streptomyces lavendulae* subsp. *lavendulae* CCM 3239 (formerly *Streptomyces aureofaciens* CCM 3239), which is responsible for the production of the angucycline-like antibiotic auricin (**1**). Preliminary characterization of **1** revealed that it possesses an aminodeoxyhexose d-forosamine and is active against Gram-positive bacteria. Here we determined the structure of **1**, finding that it possesses intriguing structural features, which distinguish it from other known angucyclines. In addition to d-forosamine, compound **1** also contains a unique, highly oxygenated aglycone similar to those of spiroketal pyranonaphthoquinones griseusins. Like several other griseusins, **1** also undergoes methanolysis and displays modest cytotoxicity against several human tumor cell lines. Moreover, the central core of the *aur1* cluster is highly similar to the partial *gris* gene cluster responsible for the biosynthesis of griseusin A and B in both the nature of the encoded proteins and the gene organization.

## 1. Introduction

Bacteria of the genus *Streptomyces* are the dominant producers of bioactive natural products with a broad range of biological activities. A large number of these products are polyketides. These structurally diverse natural compounds are synthesized by repeated decarboxylative condensation from acyl-CoA precursors by a polyketide synthase (PKS). Aromatic polyketides are synthesized by type II PKSs. Although type II PKSs produce a large repertoire of aromatic polyketides, they all belong to just a few common structural types, including pyranonaphthoquinones, tetracyclines, angucyclines, anthracyclines, tetracenomycines, aureolic acids, and pradimycin-type polyphenols [1]. Angucyclines form the largest group of aromatic polyketides. They feature a tetracyclic benz[*a*]anthracene skeleton and have a broad range of biological activities. Their biosynthesis differs from that of other aromatic polyketides by the action of a specific cyclase which closes the fourth ring, ring A, of the polyketide in an angular orientation to produce the first stable intermediate, UWM6. This intermediate is then used to produce many distinct angucyclines through the action of tailoring enzymes, with oxygenases playing a key role in generating metabolic diversity [2].

We previously identified the *aur1* gene cluster, located on the large linear plasmid pSA3239 in *Streptomyces aureofaciens* CCM 3239, which is similar to the type II PKS gene clusters for angucycline antibiotics. The *aur1* cluster is responsible for antibiotic activity against Gram-positive bacteria and the compound was named auricin [3,4,5,6]. Auricin is produced at very low levels, which hampered its purification and structural elucidation for many years. Careful investigation revealed that auricin is transiently produced during the transitional period when exponential growth gives way to stationary phase growth; after the transition, it is degraded into inactive metabolites (it is unstable at the higher pH values reached in stationary phase) [7].

Auricin’s unusual pattern of production arises from a strict, but complex regulatory mechanism, involving both feed-forward and feed-back control by auricin intermediates via several transcriptional regulators [5,8,9]. Auricin purified from this transient phase is stable in various organic solvents [7]. Preliminary characterization using ESI MS revealed that it is a polyketide, conjugated uniquely to the aminodeoxyhexose d-forosamine. The *aur1* biosynthetic cluster contains biosynthetic genes for d-forosamine, seemingly confirming its presence, but in contrast to other antibiotic clusters, these biosynthetic genes, together with several other tailoring genes, are scattered rather distantly in the *aur1* flanking regions [5,9]. The auricin production strain, *Streptomyces aureofaciens* CCM 3239 was received from the Czech Collection of Microorganisms (CCM), Brno, Czech Republic, and has been used by our group for more than 25 years [10,11,12]. Recent genomic sequence of the strain (GenBank Acc. No. CP024985) revealed that CCM 3239 was wrongly filed by CCM and is actually *Streptomyces lavendulae* subsp. *lavendulae* CCM 3239 [13]. In the present study, we report the isolation and structure elucidation of auricin (1) and its biological activity. In addition, we performed a bioinformatics analysis of the *aur1* cluster showing its high similarity to the partial *gris* cluster responsible for the biosynthesis of griseusins A and B [14].

## 2. Results and Discussion

### 2.1. Isolation and Structure Elucidation of Auricin 

To isolate auricin (**1**), the growth conditions previously published [7,9] were optimized for the highest titers of **1** and an isolation procedure using ethylacetate extraction of culture medium, a silica flesh column and preparative RP-18 HPLC was optimized to overcome problems with the stability of **1** (see Materials and Methods). This procedure resulted in the isolation of 4.5 mg of pure compound **1** from a 500 ml culture, which was verified by analytical RP-18 HPLC as described previously [7]. As previously described [7,9], **1** is a yellow compound with a molecular formula of C_28_H_31_NO_10_, based on high-resolution ESI MS, which gave an ion peak at *m*/*z* 542.20187 [M+H]^+^ (calc 542.20262 for [M+H]^+^). ESI MS/MS fragmentation of this ion produced only a single dominant signal at *m*/*z* 142.12259 [M+H]^+^, corresponding to d-forosamine. The UV spectrum of **1**, with maxima at 213, 253, and 436 nm, suggested a *peri*-hydroxy quinone chromophore, which was also confirmed by the appearance of a violet color after the addition of sodium hydroxide.

**Figure 1:**
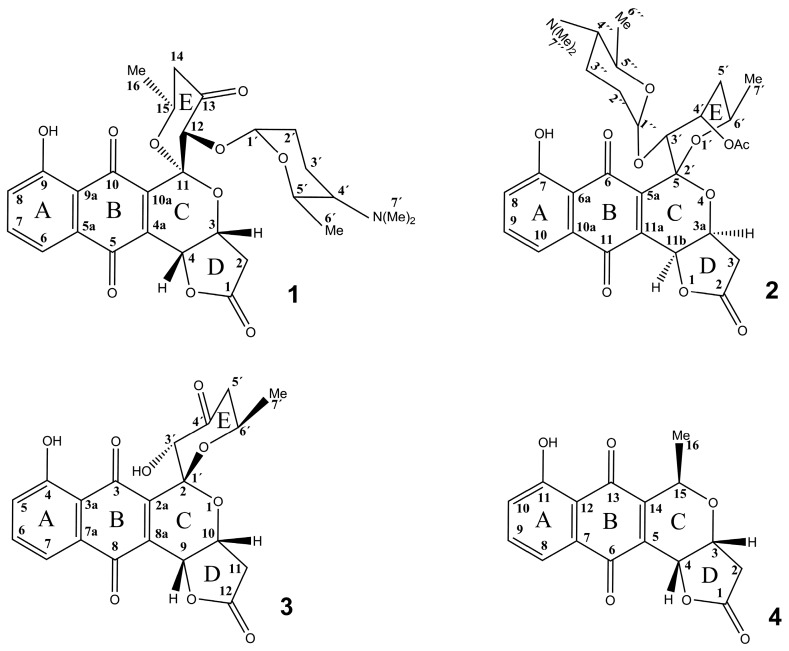
Structure of auricin (1) 3′-O-α-d-forosaminyl-(+)-griseusin A (2), 4’-dehydro-deacetylgriseusin A (3), and kalafungin (4). Numbering of positions is according to the published data for each compound.

Extensive NMR experiments (^1^H, ^13^C, COSY, TOCSY, H2BC, HMBC, and selective 1D TOCSY and 1D NOESY) were used to establish the structure of compound **1**. The NMR data of **1** are summarized in Supplementary Table S1 and its structure is shown in Figure 1. 1D and 2D ^1^H and ^13^C NMR spectra also appear in the Supplementary data.

The ^1^H NMR spectrum (Supplementary Figure S1) exhibited three aromatic methine proton signals, an OH signal at δ_H_ 12.00 (OH-9), signals of protons from three methyl, two methylene, and four aliphatic methine groups, and overlapping signals in the up-field region δ_H_ 2.1-0.5 ppm. Two dimensional (2D) ^1^H-^1^H homocorrelated COSY (Supplementary Figure S2) and TOCSY (Supplementary Figures S3 and S4) spectra revealed four coupled spin systems. The first, an ABX spin system, consisted of three aromatic protons at δ_H_ 7.692, 7.683, and 7.361, which were attributed, respectively, to H-6, H-7, and H-8 of ring A. The second spin system involved methylene protons (δ_H_ 2.884, 2.727; H-2a,b) coupled to an oxygenated methine proton (δ_H_ 5.351; H-3), which was further coupled to another proton on the oxygenated methine carbon (δ_H_ 5.360; H-4) nearby. Both methine signals (H-3 and H-4) overlapped. In the third spin system, a proton located on an oxygenated methine carbon (δ_H_ 4.562; H-15) was coupled to protons from methyl (δ_H_ 1.353; H-16) and methylene (δ_H_ 2.645, 2.604; H-14a,b) groups. The fourth spin system started with the anomeric proton H-1′ (δ_H_ 4.854) of d-forosamine showing only one three bond coupling ^3^J_H1,H2_ = 3Hz. According to the Karplus equation, this value suggests an equatorial orientation of one of two methylene protons at C2´, while the angle of the second proton with H1´should be between 120° to 60°. The COSY spectrum showed an interaction from H1´ to both methylene protons H2´a and H2´b (δ_H_ 2.05 and 1.66 ppm). The whole spin system was identified using selective 1D TOCSY after an irradiation of H1´, showing locations of H3´a and H3´b (δ_H_ 1.568 and 1.278 ppm), H4´ at δ_H_ 2.08, H5´at δ_H_ 3.108 and H6´ at δ_H_ 0.79 ppm (Supplementary Figure S4). Their carbon chemical shifts were assigned using a multiplicity-edited 2D ^1^H-^13^C heterocorrelated HSQC spectrum (Supplementary Figure S6). The d-forosamine NMR data of **1** are in very good agreement with those published for 3´O-α-d-forosaminyl- (+)-griseusin A (**3**) (Supplementary Table S2) [15].

In agreement with the C_28_H_31_NO_10_ molecular formula of compound **1** found by ESI-MS, in the ^13^C NMR spectrum (Supplementary Figure S5) twenty-seven signals (the two 7′ carbons of d-forosamine are magnetically equivalent, affording only one signal) were observed. The ^13^C spectrum also shows that a low amount of impurities (<20%) are present, which arise from the instability and the decomposition of **1**, and their signals are observed in the region δ 55–10 ppm. An edited HSQC (Supplementary Figure S6) showed that ten signals belonged to quaternary carbons. Three of them have the characteristic chemical shifts of keto-carbonyls (δ_C_ 181.45, 186.55, 202.26), and one was due to a carboxylic acid group (δ_C_ 173.75). Two oxygenated quaternary carbons had aromatic (δ_C_ 162.27) and aliphatic (δ_C_ 98.91) character. The signal at δ_C_ 162.27 was attributed to C-9 (of the A ring) due to its specific chemical shift. Of the proton-bearing carbons, three were attributed to aromatic, non-oxygenated methine carbons (δ_C_ 125.70, 137.29 and 119.59, A ring ABX spin system). The remaining signals arose from seven aliphatic methine, four methylene, and four methyl carbons. The A ring signals were assigned based on COSY, H2BC and HMBC correlations. Two-bond correlations to protonated carbons in the H2BC spectrum (Supplementary Figure S7) revealed the connectivity of the aromatic protons H-6, H-7 and H-8: H-7 (δ_H/C_ 7.683/137.29) is correlated to C-6 (δ_C_ 119.59) and C-8 (δ_C_ 125.70), while the correlations of both H-6 (δ_H/C_ 7.692/119.59) and H-8 (δ_H/C_ 7.361/125.70) led to C-7 (δ_C_ 137.29) only. Further, long-range interactions observed in the HMBC spectrum (Supplementary Figures S8 and S9) suggested the location of hydroxyl group OH-9 at C-9 and confirmed the structure of the neighboring B ring. In particular, strong correlations from OH-9 to C-8 (δ_C_ 125.70), C-9 (δ_C_ 162.27), and C-9a (δ_C_ 114.92), and weak ones to C-7 (δ_C_ 137.29) and C-10 (carbonyl, δ_C_ 186.55) were observed. The H-6 and H-7 correlations to the second carbonyl (C-5, δ_C_ 181.45) confirmed the quinone-like structure of ring B. The C-4a and C-10a assignments to signals at δ_C_ 138.05 and 140.68 ppm, respectively, were confirmed by HMBC correlations (described later). The carbon chemical shifts of the A and B rings (Supplementary Table S1) were found to agree very well with the published data for angucycline compounds containing the same structural fragment [16].

Inspection of the NMR spectra revealed the presence of methane aliphatic group structural reporter H/C signals, showing particular chemical shifts due to the near-by oxygen. They belong to the spin systems of three rings: an isolated H-12/C-12 (δ_H/C_ 5.378/80.85, ring E); H-3/C-3 signals (δ_H/C_ 5.348/71.31, ring D) and H-4/C-4 (δ_H/C_ 5.360/68.45, ring D); H-15/C-15 at δ_H/C_ 4.562/69.28 (ring E) and, finally, H-1/C-1 chemical shifts δ_H/C_ 4.856/95.94, characteristic of carbohydrates, identified as the anomeric signal of the d-forosamine. Very strong cross-peaks were observed in the HMBC spectrum from the H-2a and H-2b methylene protons (δ_H_ 2.884, 2.727) to C-1, C-3, C-4, and quaternary C-11 and weak ones to C-5, C-4a (Supplementary Figure S10). Both downfield shifted H-3 and H-4 signals correlated with carboxyl group C-1 (δ_C_ 173.75), however, the H-3/C-4a and H-4/C-10a correlations were the most intense from these protons (Supplementary Figure S11). All these correlations were important for elucidating the structure of the C ring. The data obtained (Supplementary Table S1) confirmed that the -^1^COO-^2^CH_2_-^3^CH(O)-^4^CH(O)- fragment is located in close proximity to the quaternary C-4a and C-5 carbonyl (ring B). Furthermore, the fact that the H-4/C-4 atoms are oxygenated indicated that this structural fragment forms the ring D by joining C-4 and carboxyl C-1 via a carboxylic oxygen. In addition, the strong H2-a/C-11 correlation confirmed closure of the 6-membered ring C via oxygen at C-3 to C-11 (δ_C_ 98.91).

NMR data also showed that the oxygenated H-15/C-15 signals (δ_H/C_ 4.562/69.28) belong to another structural fragment, which also includes H-14 and H-16. These protons show multiple mutual HMBC correlations, but all three correlated with the C-13 carbonyl (δ_C_ 202.26), resulting in a second fragment with a -^13^CO-^14^CH_2_-^15^CH(O)-^16^CH_3_ structure. The H-14/C-12 correlation together with that of H-12 to C-11 and C-10a (Supplementary Figure S10), and the fact that CH-15 is oxygenated, indicates that the 6-membered ring E must be closed at quaternary C-11. The correlations of H-15 and H-16 to the quaternary C-11 supported such structure. The unusual chemical shifts of the isolated oxygenated H-12/C-12 signals indicated that the aglycone is linked to d-forosamine at C-12. This conclusion was supported by a through-space interaction between H-12 and H-1´in the NOESY and ROESY spectra (Supplementary Figures S12 and S13) and a C1´/C12 correlation in the HMBC (Supplementary Figure S10). The results of the HMBC, COSY, and NOESY experiments on **1** are summarized in Supplementary Figure S14 and Table S1. Supplementary Figure S15 shows a model of the 3D structure of compound **1** prepared with Chem3D Pro using a simple MM2 force field energy optimization. In this model, the NOE interactions observed in the NMR spectra could be observed. The absolute configurations of the chiral centers are C11(S), C12(S), C1´(R) and C4´(S). The characteristic vibrations found in the infrared spectrum also support the proposed structure of **1** (Supplementary Figure S16 and Table S3).

In addition to the presence of d-forosamine, which is unique among the known angucyclines [9], the resulting structure of **1** also has an intriguing, highly oxygenated aglycone. The *peri*-hydroxy quinone rings A and B are characteristic of these types of compounds (Figure 1), however, to our knowledge, the three lactone rings, including the [6,6] spiroketal moiety of rings C and E, are unique among angucyclines. Interestingly, the structure of the auricin aglycone strikingly resembles those of the griseusins, e.g., 3′-O-α-d-forosaminyl-(+)-griseusin A (**2**) [15] and 4´-dehydro-deacetylgriseusin A (**3**) [17] (Figure 1). However, griseusins are a subgroup of the pyranonaphthoquinone family of aromatic polyketides, which contain a [6,6] spiroketal ring system fused to a juglone moiety (Figure 1). Like **1**, a γ-lactone moiety is present in both **2** and **3**, as well as in some other members of the pyranonaphthoquinone family, e.g., kalafungin (**4**) [18]. In accord with this resemblance, a simple comparison of the ^1^H and ^13^C NMR data for **1**, **2**, and **3** showed that the shifts of the of aglycone part of these molecules are nearly identical, including those of the critical spiroketal two-oxygen-bearing quaternary carbon (Supplementary Table S2). The structural γ-lactone motif of ring D is identical in all three compounds. In **1** and **2** the aglycone is different at position 13 (carbonyl) and 4´(acetyl), respectively, influencing the chemical shifts of H-12 in **1** and H-3´in **2**. In **1** and **3** the aglycone structures are identical, but d-forosamine is missing at position 3´ in the E ring of **3**. These small differences are also reflected in the slight variations of the chemical shifts of most signals. An important change was observed in the γ-lactone (ring D), particularly at H-3 in **1** and H-10 in **3** (Supplementary Figure S14), probably caused by the interaction of H-3 (ring D) with H-3´ of d-forosamine, which is absent in **3**. The differences in the chemical shift of quaternary C-11 might be caused by the different absolute configurations of this chiral center, S in **1** and R in **3**. As mentioned above, the spectral data of d-forosamine in both **1** and **2** were consistently nearly identical, and both the UV and IR spectra of **1** and **2** are also very similar [15]. The data obtained surprisingly suggest that compound **1**, previously thought to be an angucycline-like antibiotic based on genetic data [3,4,5,6,7,8,9], belongs instead to the pyranonaphthoquinone subfamily of griseusins. Based on the above results, the structure of **1** corresponds to the systematic name (2’S,3aR,3’S,6’S,11bR)-3’-(((2R,6R)- 5-(dimethylamino)-6-methyltetrahydro-2H-pyran-2-yl)oxy)-7-hydroxy-6’-methyl-3,3a,5’,6’-tetra- hydrospiro[benzo[g]furo[3,2-c]isochromene-5,2’-pyran]-2,4’,6,11(3’H,11bH)-tetraone. It is a new O-α-d-forosaminyl-dehydro-deacetylgriseusin. 

### 2.2. Methanolysis of Auricin

We encountered difficulties purifying **1** in silica columns when using methanol as a solvent. Compound **1** is always degraded under these conditions. This problem was overcome by using ethanol as a solvent as described in the purification procedure. As described previously [6], purified compound **1** is stable for at least 6 days in acetonitrile and ethanol. Therefore, we similarly tested the stability of purified **1** in methanol with and without silica. HPLC analysis showed that **1** (Rt = 8.6 min) is gradually converted to a new peak at Rt = 7.2 min. This conversion was significantly accelerated by the presence of silica, and after 24 h almost all **1** was converted to the new peak (Supplementary Figure S17a). This new peak was isolated, and a high-resolution ESI MS analysis produced a molecular ion [M+H]^+^ at *m*/*z* 574.2246, which corresponds to a molecular formula of C_29_H_35_NO_11_ (calc 574.2288 for [M+H]^+^) (Supplementary Figure S17b). Thus, there is an increase in mass of 32 amu, indicating the addition of methanol (methanolysis) to **1**. ESI MS/MS fragmentation of this ion was performed to determine the position of the methanol addition. As described previously [9], the molecular ion [M+H]^+^ of **1** (*m*/*z* = 542.2043 [M+H]^+^) produces only a single dominant signal at *m*/*z* 142.1 [M+H]^+^ after ESI MS/MS fragmentation, corresponding to d-forosamine; however, the molecular ion [M+H]^+^ (*m*/*z* = 574.2246) of the new compound produced an identical d-forosamine ion. This indicates that the reaction occurs in the aglycone moiety of **1**.

Semisynthetic studies with several griseusins, including **3**, provided new derivatives by opening the γ-lactone ring D using acid-catalyzed methanolysis. Interestingly, this reaction was also observed as an unwanted side reaction during the chromatographic isolation of **3**. In this way 4’-dehydro-9-hydroxy-deacetylgriseusin B methyl ester was obtained from **3** [17]. The difference in mass of both compounds is also 32 amu, identical to our case. Interestingly, they also used a silica column and methanol as a solvent for the isolation of griseusins, conditions that are not suitable for the isolation of compound **1**, as stated above. Since **3** is almost identical to the aglycone of **1** (Figure 1, Supplementary Table S2), we conclude that an identical methanolysis likely occurs, in which the γ-lactone ring D in **1** is opened, resulting in methoxyauricin (Supplementary Figure S17c). The catalytic effect of silica on this methanolysis no doubt arises from the weak acidic properties of silica. 

### 2.3. Bioinformatic Analysis of the Auricin Cluster 

**Figure 2:**
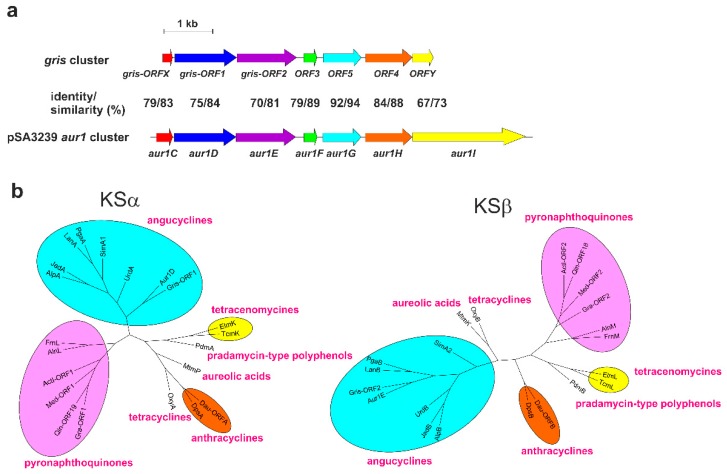
(**a**). Comparison of the gene organization of the core auricin *aur1* cluster from pSA3239 [5] with the griseusin biosynthetic gene cluster of *S. griseus* K-63 [14]. Homologous genes (together with their identity and similarity) are represented by arrows with identical colors. (**b**) Phylogenetic trees of the ketosynthase α (KSα) and ketosynthase β (KSβ) involved in the biosynthesis of representative groups of aromatic polyketides. Trees were constructed using the Neighbour Joining method [21], based on a comparison of KSα and KSβ *from S. lavendulae subsp. lavendulae* CCM 3239 with the griseusin KSα and KSβ from *S. griseus* K-63 [14] and several representative KSα and KSβ proteins from the main groups of aromatic polyketides. The protein alignments and their corresponding descriptions are given in Supplementary Figures S19 and S20.

Since the structure of **1** suggests, surprisingly that **1**, previously expected to be an angucycline-like antibiotic, actually belongs to the pyranonaphthoquinone subgroup of griseusins, we thoroughly analyzed the *aur1* cluster and its flanking regions in the large linear plasmid pSA3239 located in *S. lavendulae* subsp. *lavendulae* CCM 3239. The central part of the *aur1* cluster for **1** contains the initial biosynthetic genes *aur1C*, *aur1D*, *aur1E*, *aur1F*, *aur1G*, *aur1H*, which are homologous to the angucycline genes encoding a cyclase (CYC), ketosynthase α (KSα), ketosynthase β (KSβ), acyl carrier protein (ACP), ketoreductase (KR), and aromatase (ARO), respectively. In addition, the organization of these genes, including the angucycline-specific CYC gene *aur1C*, is strictly conserved in all angucycline clusters (Supplementary Figure S18) [2,5]. Deleting the initial biosynthetic gene of **1**, *aur1D*, which encodes KSα [3], and the *aur1* cluster from *sa22* to *aur1V* [4], results in the absence of **1**, thus clearly confirming the role of these *aur1* core genes in the biosynthesis of **1**. Only one partial biosynthetic gene cluster (*gris*) for the biosynthesis of griseusin A and B in *S. griseus* K-63 [14] has been described in the literature or databases. Interestingly, it is highly similar both in its gene organization and in the proteins it encodes to the core *aur1* gene cluster (Figure 2a). Moreover, our analysis of adjacent *gris* regions revealed two new incomplete ORFs, Gris-ORFX and Gris-ORFY, which were highly similar to Aur1C CYC and Aur1I oxygenase, respectively (Figure 2a, Supplementary Figures S21 and S22). 

Interestingly, although griseusins clearly form a subgroup of pyranonaphthoquinones on the basis of their structures [19], a phylogenetic analysis of their aromatic polyketide synthases KSα and KSβ localized both of the initial biosynthetic proteins for griseusin (Gris-ORF1 and Gris-ORF2) to the angucyclines clade and not the pyranonaphthoquionoines clade [20]. We performed a similar phylogenetic analysis with the KSα (Aur1D) and KSβ (Aur1E) of **1** with the KSα and KSβ (Gris-ORF1, Gris-ORF2) of griseusin and several representative KSα and KSβ proteins from the major groups of aromatic polyketides. Both griseusin KSα and KSβ were clearly placed in the angucycline clade and they most resembled the KSα and KSβ of **1** of all the angucyclines (Figure 2b).

These genetic data, together with the structural analysis, therefore confirm that the *aur1* cluster responsible for biosynthesis of **1** is involved in the biosynthesis of a griseusin-like compound and that both **1** and griseusins are likely to be synthesized in an angucycline-like biosynthetic pathway, at least in the early stages. Thus, the *aur1* cluster (Supplementary Figure S18) from *S. lavendulae* subsp. *lavendulae* CCM 3239 represents the first complete biosynthetic gene cluster for a griseusin. To avoid confusion, we would like to continue using the original name for **1** in the future, since 14 papers have been published on its genetic and regulatory properties. The structure of **1** also clarified a number of the surprising properties of the *aur1* cluster. Although, as described above, its core is similar to the angucycline biosynthetic gene clusters, unlike these clusters, it contains many putative tailoring biosynthetic genes encoding oxygenase and dehydrogenase homologs scattered in areas quite distant from the *aur1* core. In addition, we recently found that several d-forosamine biosynthetic genes also appear in in a region rather distant from the core of the *aur1* cluster (Supplementary Figure S18) [9]. Some of these scattered biosynthetic genes are similar to pyranonaphtoquinones biosynthetic genes. They are absent from other angucycline gene clusters and were found to be under the control of auricin-specific positive regulators [5,9]. They can, therefore, participate in the biosynthesis of **1** and are responsible for this intriguing structure. Deletion analysis of these genes and further studies on the biosynthesis of **1** are in progress.

### 2.4. Biological Activity of Auricin

Previous studies revealed that compound **1** is active against Gram-positive bacteria [3,7,9]. In the present study we determined the MICs for *Bacillus subtilis* and *Staphyloccocus aureus* Newman which were 4.6 μM and 9.2 μM, respectively. In addition, **1** also displayed modest cytotoxicity against several human cancer cell lines (Table 1). However, due to its instability at neutral pH [7], it seems likely that **1** progressively degraded during these cytotoxic assays and its actual cytotoxicity values are likely to be even higher. Like **1**, griseusins are particularly active against Gram-positive bacteria [15,22,23,24], and many of them are very effective against human cancer cell lines [17,22,25]. The biological activities of **1** are therefore also similar to griseusins. However, no griseusin derivative has been reported to have this interesting instability at neutral and higher pH.

## 3. Materials and Methods

### 3.1. Strain and Cultivation

The *Streptomyces aureofaciens* CCM 3239 wild-type strain was received from the Czech Collection of Microorganisms (CCM), Brno, Czech Republic [7,9]. However, its recent genomic sequence (GenBank Acc. No. CP024985) revealed that CCM 3239 was wrongly filed by CCM and the organism is actually *Streptomyces lavendulae* subsp. *lavendulae* CCM 3239 [13]. This was subsequently corrected by CCM. For sporulation, *S. lavendulae* subsp. *lavendulae* CCM 3239 was grown at 28 °C on modified solid Bennet medium (Difco Tryptone 2 g, Difco Beef extract 1 g, Difco Yeast extract 1 g, maltose 10 g, Difco agar 20 g, in 1 l distilled water, pH 7.2) [26] for 10 days. Fresh spores were collected from the medium with water, twice filtered through cotton wool, centrifuged, suspended in small aliquots in 20% glycerol, and stored at −80°C. Growth of the *S. lavendulae* subsp. *lavendulae* CCM 3239 strain was carried out in liquid-rich Bennet medium as described in [26]. For isolation of **1** from liquid-grown cultures, *S. lavendulae* subsp. *lavendulae* CCM 3239 spores (4 × 10^9^ c.f.u) were inoculated into 50 mL Bennet medium (Difco Tryptone 2 g, Difco Beef extract 1 g, Difco Yeast extract 1 g, maltose 10 g, in 1 l tap water, pH 7.2) in 500 mL Erlenmeyer flasks, and the cultures were cultivated on a rotary shaker at 270 r.p.m. at 28 °C for 14 h.

### 3.2. Extraction and Purification of Auricin

The 500 ml culture of *S. lavendulae* subsp. *lavendulae* CCM 3239 was filtered to remove mycelium. The filtrate was extracted twice with the same volume of HPLC-grade ethyl acetate. Residual water was removed with sodium sulfate and sodium chloride, and the extract was evaporated under vacuum. The yellow-brown pellet remaining was dissolved in 8 mL of 9:1 dichloromethane: ethanol and purified through a 20 mL silica column equilibrated with the same solvent. The 200 mL first fraction containing yellow **1** was concentrated and dissolved in 2 mL 50% ethanol. Subsequent fractions of 0.25 mL were purified by semi-preparative reverse-phase HPLC using an OmniSpher C18 column (250 × 10 mm) (Varian, Lake Forest, CA) with an isocratic elution of 25% acetonitrile and 0.5% acetic acid in water at a 4 mL/min flow rate (VIS detection at 450 nm). The major peak was collected to yield 4.5 mg of **1**. The purity and yield of **1** were estimated by analytical HPLC as described previously [7,9].

### 3.3. Mass Spectroscopy

High-resolution ESI mass determination was performed using an Orbitrap Velos PRO spectrophotometer (Thermi Fisher Scientific).

### 3.4. NMR Spectroscopy

The NMR spectra of **1** were measured in 3 mm sample tubes in deuterated chloroform (CDCl_3_) at 25 °C on a Bruker AVANCE III HDX 600 MHz NMR spectrometer equipped with a liquid He cooled Triple inverse TCI H-C/N-D-05-Z cryo probe. 3-(Trimethylsilyl)propionic-2,2,3,3-d4 acid sodium salt (TSPd_4_) was used for chemical shift calibration (δ_H/C_ 0/0 ppm). The following 1D and 2D pulse sequences from the Bruker pulse sequence library were used: 2D ^1^H-^1^H-homonuclear correlated experiments COSY (cosygpmfppqf) and TOCSY (mlevphpr), 2D ^1^H-^13^C heteronuclear correlated HSQC (hsqcedetgpsisp2.3), HMBC (hmbcgplpndqf), H2BC (h2bcedetgppl3), selective HMBC (shmbcctetgpl2nd), and 1D NOESY (selnogp) with selective excitations. The ^1^H and ^13^C chemical shifts and correlations observed in the NMR spectra between protons and carbons are summarized in Table S1. The main NMR data of **1** are the following: ^1^H NMR (*δ*, CDCl_3_) 2.884 (dd, *J* = 5.2 Hz, 18.1 Hz, 1H, H-2a), 2.727 (d, *J* = 18.1 Hz, 1H, H-2b), 5.351 (dd, *J*, 1H, H-3), 5.360 (d, *J*, 1H, H-4), 7.692 (*J*, 1H, H-6), 7.683 (*J*, 1H, H-7), 7.361 (dd, *J* = 4.6, 5.2 Hz, 1H, H-8), 5.382 (s, 1H, H-12), 2.645 (dd, *J* = 10.7, 13.9 Hz, 1H, H-14a), 2.604 (dd, *J* = 3.3, 13.9 Hz, 1H, H-14b), 4.562 (m, *J* = 3.3, 6.0, 10.5 Hz, 1H, H-15), 1.353 (d, *J* = 6.4 Hz, 3H, H-16), 12.0 (s, 1H, OH-9), 4.854 (d, *J* = 3.0 Hz, 1H, H-1’), 2.041 (m, 1H, H-2’a), 1.661 (m, 1H, H-2’b), 1.576 (m, 1H, H-3’a), 1.274 (m, 1H, H-3’b), 2.08 (m, 1H, H-4’), 3.111 (m, 1H, H-5’), 0.792 (d, *J* = 6.2 Hz, 3H, H-6’), 1.835 (s, 6H, H-7’); ^13^C NMR (*δ*, CDCl_3_) 173.75 (C-1), 37.39 (C-2), 71.31 (C-3), 68.45 (C-4), 138.05 (C-4a), 181.45 (C-5), 131.06 (C-5a), 119.59 (C-6), 137.29 (C-7), 125.70 (C-8), 162.27 (C-9), 114.92 (C-9a), 186.55 (C-10), 140.68 (C-10a), 98.91 (C-11), 80.85 (C-12), 202.26 (C-13), 48.88 (C-14), 69.28 (C-15), 21.55 (C-16), 95.94 (C-1’), 29.40 (C-2’), 14.70 (C-3’), 64.62 (C-4’), 68.30 (C-5’), 17.74 (C-6’), 39.91 (C7’).

### 3.5. Infrared Spectrometry

Fourier-transform infrared (FTIR) spectra were measured with a Nicolet iS50 FT-IR spectrometer (Thermo Scientific, USA) equipped with a DTGS detector and Omnic 9.0 software. The spectrum was collected in the middle region from 4000 to 400 cm^−1^ at a resolution of 4 cm^−1^, with 64 scans. The Diamond ATR accessory for solid-state measurements was used. 

### 3.6. MIC Determination

The minimum inhibitory concentration (MIC) of **1** against Gram-positive bacteria was determined by a conventional broth microdilution assay in 96 well plates. Compound **1** and tetracycline as a positive control were dissolved in 96% ethanol to a final concentration of 1 mg/mL. *Bacillus subtilis* PY79 and *Staphyloccocus aureus* Newman were grown in liquid nutrient broth medium (NB, Difco) for 16 h at 37 °C. The microbial cultures were diluted in fresh NB to a final concentration of 10^7^ CFU/mL. The bacterial samples were then mixed with serial dilutions of the test compounds and 150 μL aliquots were dispensed into 96-well plates. The final concentrations of the compounds were 20, 10, 5, 2.5, 1.25, 0.625, and 0.3125 μg/mL. As a negative control, the 96% ethanol was similarly diluted to produce ethanol concentrations equivalent to those that resulted from dilution of the inhibitory compounds. Plates were incubated at 250 rpm and 37 °C and A_600_ against NB were measured in 1 h intervals in a Synergy HT (BioTec) microplate reader. All experiments were performed in triplicate.

### 3.7. Cytotoxicity Assay

The cytotoxicity of **1** was evaluated in tumor cell lines with the MTT assay [27]. Human ovarian carcinoma cell lines A2780, cisplatin-resistant cells A2780/CP and breast cancer cell lines MDA-MB-231 and MCF-7 were routinely cultured in RPMI 1640 (Gibco) medium supplemented with 10% heat-inactivated FCS (Gibco), 2 mM l-glutamine, 100 μg/mL penicillin and 50 μg/mL streptomycin (PAN-Biotech GmbH). The cell cultures were passaged twice a week after reaching a cell density of 0.8–1.0 × 10^6^ cells/mL. Cells were plated at 1 × 10^4^cells/well on the day before treatment and exposed to various concentrations of **1** for the respective time indicated. The stock solution of **1** was 1 mg/mL in 96% ethanol, doxorubicin in the same concentration was used as a positive control, and an equal volume of ethanol was added as a solvent control. The cells were seeded at 1 × 10^4^ cell density in 96-well culture plates. Each dose of the compound was tested in triplicates. Cell proliferation kinetics were measured in an IncuCyte^TM^ Kinetic Imaging System (Essen BioScience, UK). After 72 h, cells were incubated with 50 μL of MTT reagent (Sigma Chemical Co.) (1 mg/mL) and left in the dark at 37 °C for an additional 4 h. Thereafter, the medium was removed, the formazan crystals were dissolved in 150 μL of DMSO, and the absorbance was measured at 540 and 690 nm in an xMark™ Microplate Spectrophotometer (Bio-Rad Laboratories, Inc.). The concentration of drug that inhibited cell survival to 50% (IC_50_) was determined using Calcusyn software (version 1.1, Biosoft).

## 4. Conclusions

The structure of the previously characterized angucycline-like antibiotic auricin (**1**) was elucidated by 1D, 2D NMR spectroscopy and IR spectroscopy. Its structure has interesting properties, distinguishing it from other known angucyclines. In addition to d-forosamine, it contains a unique aglycone, which is highly similar to the spiroketal pyranonaphthoquinones griseusins. Like several other griseusins, **1** also undergoes silica-catalyzed methanolysis by opening the γ-lactone ring D, resulting in methoxyauricin. Like griseusins, **1** is particularly effective against Gram-positive bacteria and exhibits cytotoxicity against several human tumor cell lines. A bioinformatics analysis of the *aur1* cluster responsible for the biosynthesis of **1** showed that its central part is highly similar both in its organization and in the proteins it encodes to the partial *gris* gene cluster responsible for the biosynthesis of griseusin A and B. The *aur1* cluster from *S. lavendulae* subsp. *lavendulae* CCM 3239, therefore, represents the first complete gene cluster for griseusins.

## Figures and Tables

**Table 1:** Inhibition, IC_50_ (μM)^/1^, of human cancer cell lines by auricin (**1**).

Cancer Cell Line^/2^	IC_50_ (μM)
A2788	1.05
A2780/CP	0.7
MDA-MB-231	4.19
MCF-7	2.8

^/1^ − Dose required to inhibit growth to 50% of that of the untreated cells. ^/2^ – A2788 = ovarian carcinoma; A2788/CP = cisplatin resistant ovarian carcinoma; MDA-MB-231 = breast carcinoma; MCF-7 = breast carcinoma.

## Supplementary Materials

The following are available online at https://www.mdpi.com/2079-6382/8/3/102/s1, Figure S1: ^1^H NMR spectrum of auricin (**1**); Figure S2: ^1^H-^1^H COSY spectrum of **1**; Figure S3: ^1^H-^1^H TOCSY spectrum of **1; Figure S4**: Selective 1D TOCSY spectrum of **1**; Figure S5: ^13^C NMR spectrum of **1**; Figure S6: ^1^H-^13^C HSQC spectrum of **1**; Figure S7: ^1^H-^13^C H2BC spectrum of **1**; Figure S8: ^1^H-^13^C HMBC spectrum of **1**; Figure S9: ^1^H-^13^C HMBC spectrum of **1**; Figure S10: ^1^H-^13^C HMBC spectrum of **1**; Figure S11: ^1^H-^13^C HMBC spectrum of **1**; Figure S12: ^1^H-^1^H NOESY spectrum of **1**; Figure S13: Selective 1D NOESY spectrum of **1**; Figure S14: The most important correlations of **1**; Figure S15: 3D structure of **1**; Figure S16: ATR Infrared spectrum of **1**; Figure S17: Analysis of conversion of **1** to methoxyauricin; Figure S18: Genetic organization of the auricin (**1**) *aur1* cluster; Figure S19: Comparison of KSα (Aur1D) *from S. lavendulae subsp. lavendulae* CCM 3239 with the griseusin KSα from *S. griseus* K-63 (Gris-ORF1) and several representative KSα proteins from main groups of aromatic polyketides; Figure S20: Comparison of KSβ (Aur1E) *from S. lavendulae subsp. lavendulae* CCM 3239 with the griseusin KSα from *S. griseus* K-63 (Gris-ORF2) and several representative KSα from main groups of aromatic polyketides; Figure S21: Comparison of auricin CYC (Aur1C) *from S. lavendulae subsp. lavendulae* CCM 3239 with the griseusin partial CYC from *S. griseus* K-63 (Gris-ORFX); Figure S22: Comparison of auricin oxygenase Aur1I *from S. lavendulae subsp. lavendulae* CCM 3239 with the griseusin partial oxygenase from *S. griseus* K-63 (Gris-ORFY); Table S1: ^1^H and ^13^C NMR data of **1**; Table S2: Comparison of ^1^H and ^13^C NMR data for **1**, **2** and **3**; Table S3: Characteristic FTIR bands identified in the spectrum of **1**.
