# Supplementary material for: A Structural Analysis of the Angucycline-Like Antibiotic Auricin from Streptomyces lavendulae Subsp. Lavendulae CCM 3239 Revealed Its High Similarity to Griseusins
Source: Antibiotics (Basel). 2019 Jul 25;8(3):102. doi: 10.3390/antibiotics8030102 (PMC6784081; doi:10.3390/antibiotics8030102)
Supplement: Supplementary file 1 [file antibiotics-08-00102-s001.pdf]

Supporting information for:

The structural analysis of the angucycline-like antibiotic auricin from *Streptomyces lavendulae* subsp. *lavendulae* CCM 3239 revealed its high similarity to griseusins

Maria Matulova[1#], Lubomira Feckova[2#], Renata Novakova[2], Erik Mingyar[2], Dominika Csolleiova[2], Martina Zduriencikova[1], Jan Sedlak[3], Vladimir Patoprsty[1], Vlasta Sasinkova[1], Iveta Uhliarikova[1], Beatrica Sevcikova[2], Bronislava Rezuchova[2], Dagmar Homerova[2], Jan Kormanec[2*]

[1] Institute of Chemistry, Slovak Academy of Sciences, 845 38 Bratislava, Slovak Republic; e-mail@e-mail.com
[2] Institute of Molecular Biology, Slovak Academy of Sciences, 845 51 Bratislava, Slovak Republic; maria.matulova@savba.sk
[3] Cancer Research Institute BMC, Slovak Academy of Sciences, 845 05 Bratislava, Slovak Republic; jan.sedlak@savba.sk
[#] These authors contributed equally to this work
* Correspondence: jan.kormanec@savba.sk; Tel.: +421259307419 (J.K.)

Table of Contents

Figure S1. ^{1}H NMR spectrum of auricin (**1**) in $CDCl_3$ at 25°C. ……..……………………………..3
Figure S2. ^{1}H-^{1}H COSY spectrum of **1** in $CDCl_3$ at 25°C.............…………………………..3
Figure S3. ^{1}H-^{1}H TOCSY spectrum of **1** in $CDCl_3$ at 25°C.......………………..........…………4
Figure S4. Selective 1D TOCSY spectrum of **1** in $CDCl_3$ at 25°C...…………………...........….4
Figure S5. ^{13}C NMR spectrum of **1** in $CDCl_3$ at 25°C. ……...........……………………………5
Figure S6. ^{1}H-^{13}C HSQC spectrum of **1** in $CDCl_3$ at 25°C. ....…………………………..5
Figure S7. ^{1}H-^{13}C H2BC spectrum of **1** in $CDCl_3$ at 25°C.............……………………………..6
Figure S8. ^{1}H-^{13}C HMBC spectrum of **1** in $CDCl_3$ at 25°C. Detail 1............……………………..6
Figure S9. ^{1}H-^{13}C HMBC spectrum of **1** in $CDCl_3$ at 25°C. Detail 2...........……………………...7
Figure S10. ^{1}H-^{13}C HMBC spectrum of **1** in $CDCl_3$ at 25°C. Detail 3..........…………………….7
Figure S11. ^{1}H-^{13}C HMBC spectrum of **1** in $CDCl_3$ at 25°C. Detail 4..........…………………….8
Figure S12. ^{1}H-^{1}H NOESY spectrum of **1** in $CDCl_3$ at 25°C..........…………………………...…8
Figure S13. Selective 1D NOESY spectrum of **1** in $CDCl_3$ at 25°C..........……………………….9
Figure S14. The most important correlations of **1**........………………………………………....9
Table S1. ^{1}H and ^{13}C NMR data of **1**…..……………………........... 10
Table S2. Comparison of ^{1}H and ^{13}C NMR data for **1, 2** and **3**..........……………………........... 11
Figure S15. 3D structure of **1**.............................…………………………………………….....12
Figure S16. ATR Infrared spectrum of **1**……...........………………………………… ……….12
Table S3. Characteristic FTIR bands identified in the spectrum of **1**………………………..........13
Figure S17. Analysis of conversion of **1** to methoxyauricin………………………...……….14
Figure S18. Genetic organisation of the auricin (**1**) *aur1* cluster ……………………...……….15
Figure S19. Comparison of auricin KSα (Aur1D) with griseusin KSα (Gris-ORF1)
and several representative KSα proteins from main groups of aromatic polyketides..................16
Figure S20. Comparison of auricin KSβ (Aur1E) with griseusin KSβ (Gris-ORF2)
and several representative KSβ proteins from main groups of aromatic polyketides..................19
Figure S21. Comparison of auricin CYC Aur1C with griseusin partial CYC.............................22
Figure S22. Comparison of auricin oxygenase Aur1C with griseusin partial oxygenase............22

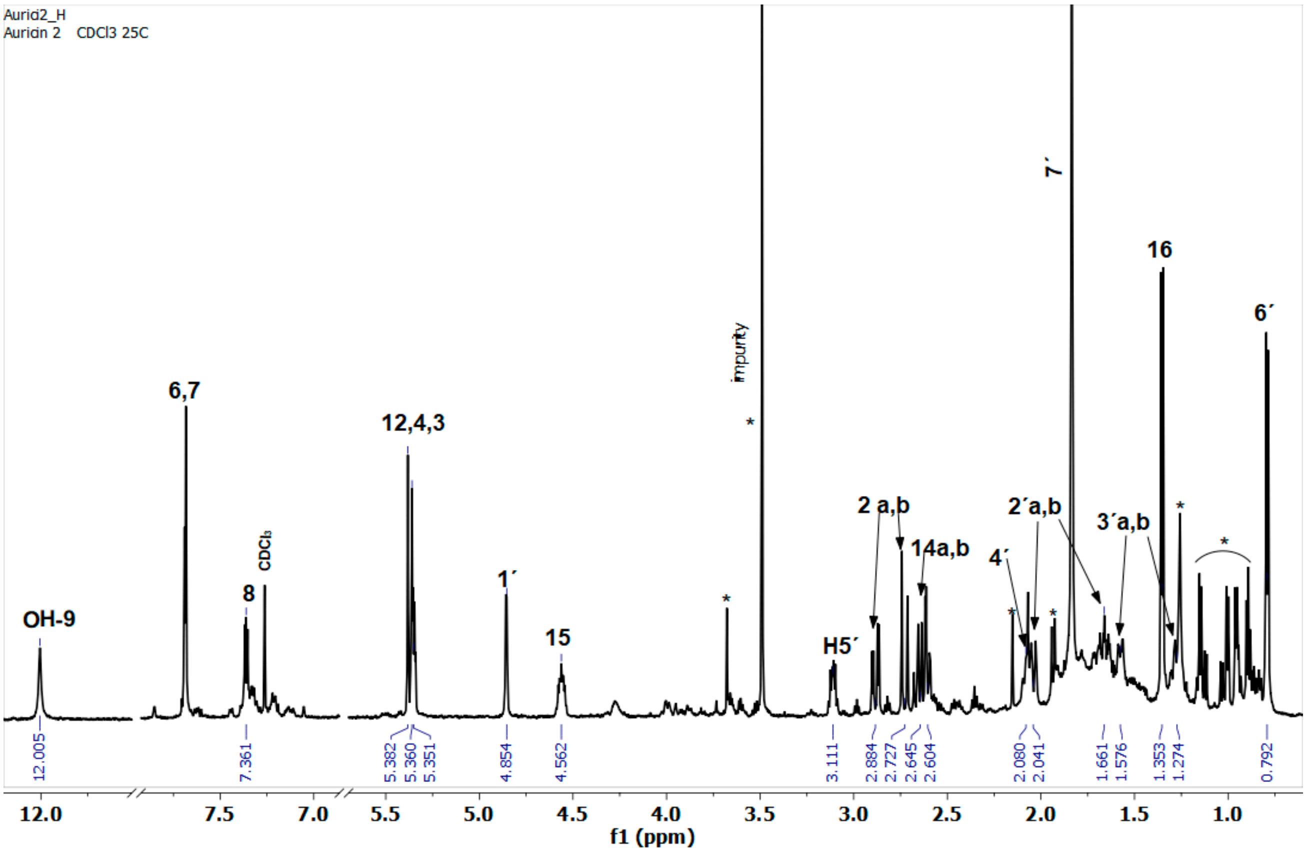

Figure S1. ^{1}H NMR spectrum of auricin (**1**) in $CDCl_3$ at 25°C.

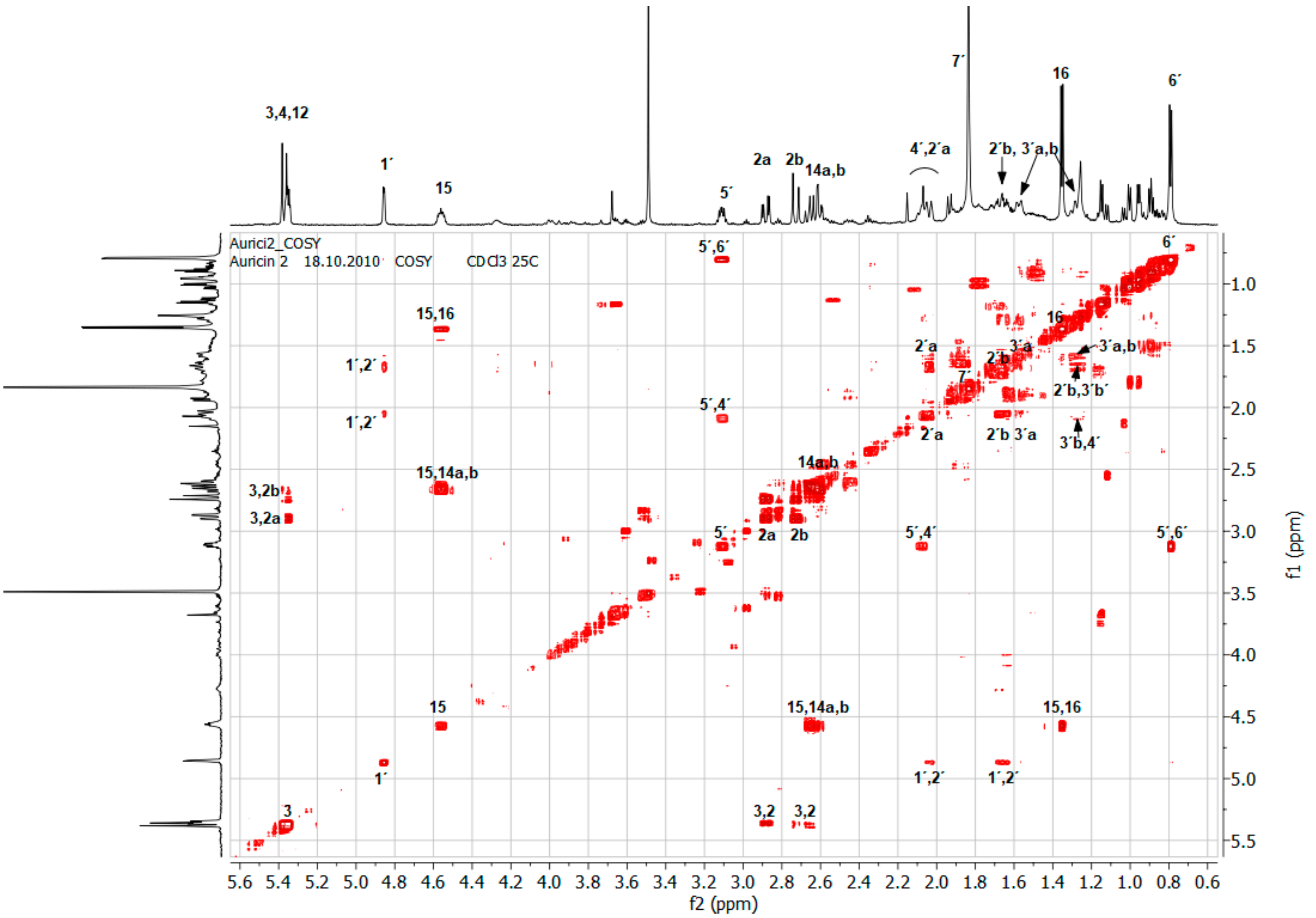

Figure S2. ^{1}H-^{1}H homocorrelated COSY spectrum of **1** in $CDCl_3$ at 25°C.

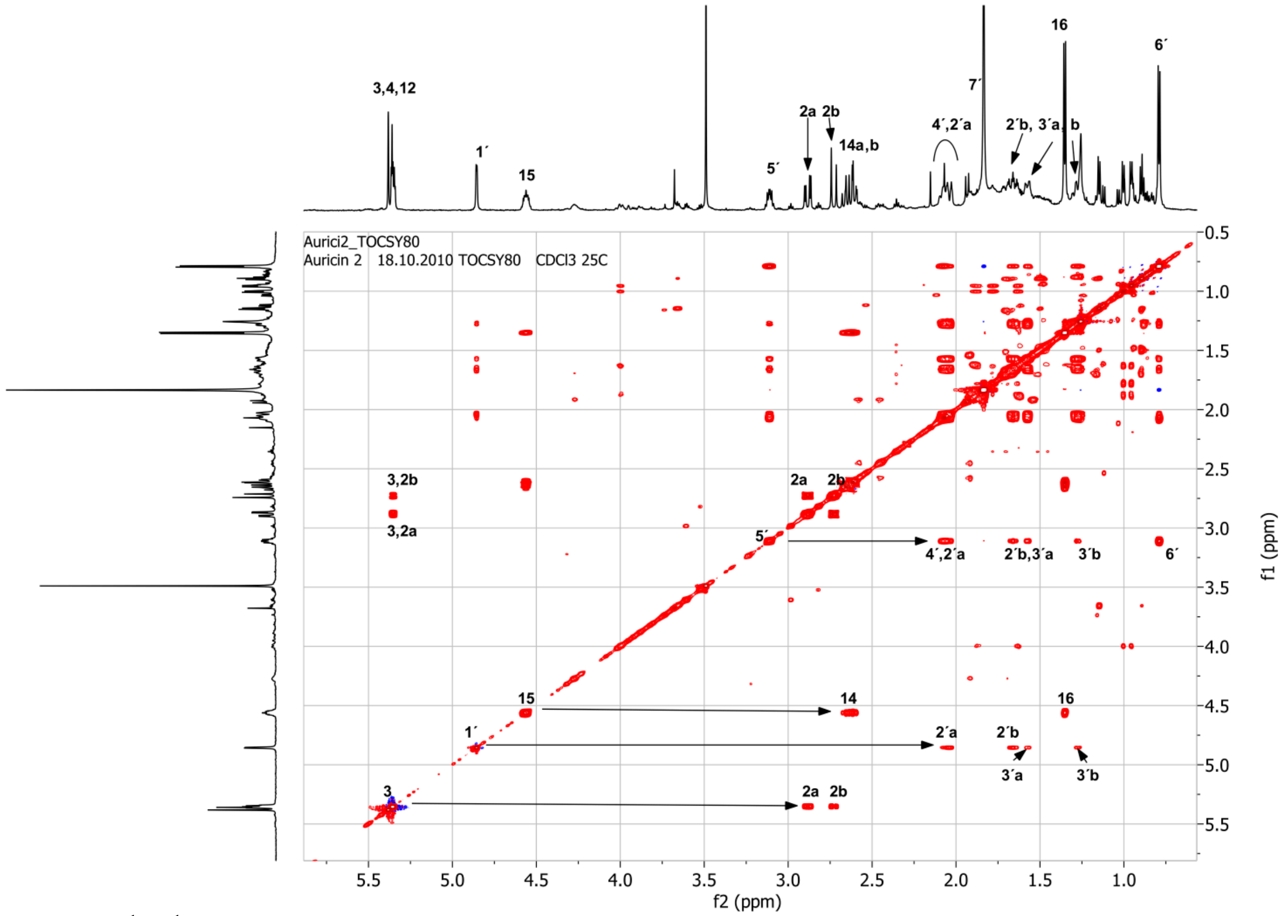

Figure S3. ^{1}H-^{1}H homocorrelated TOCSY spectrum (80 ms mixing time) of **1** in $CDCl_3$ at 25°C.

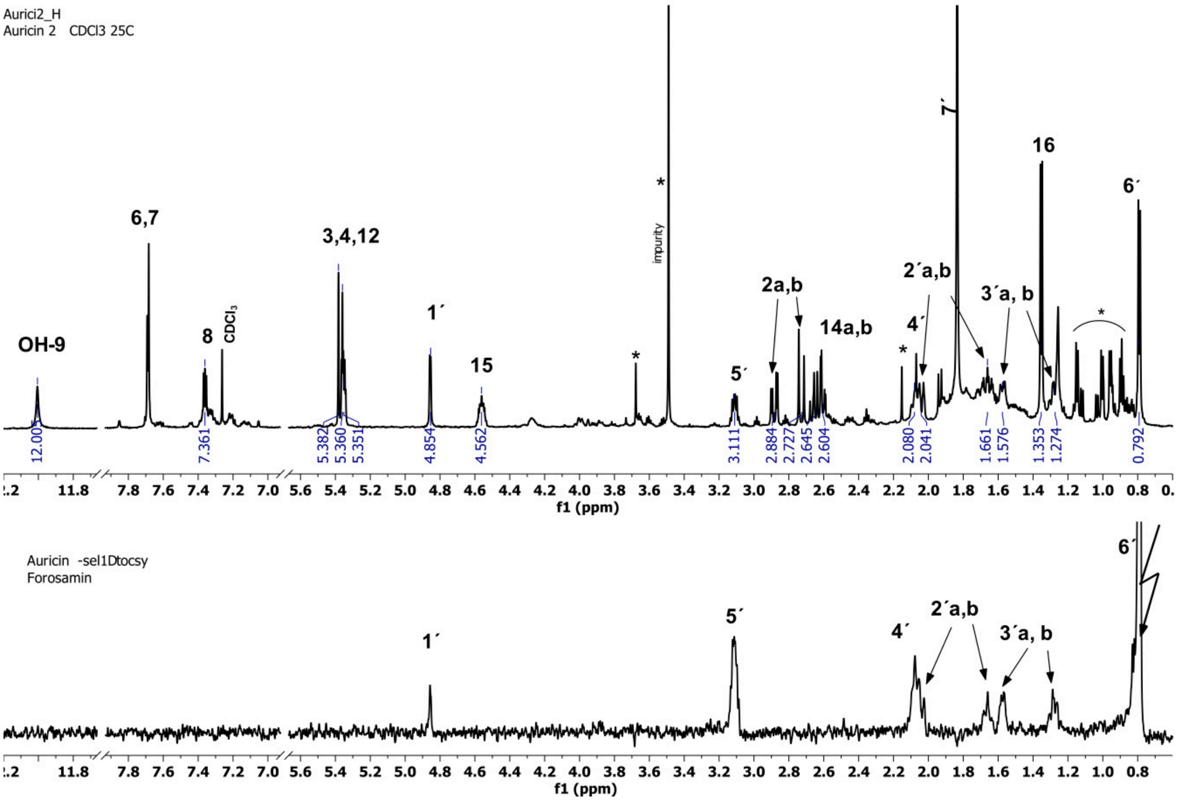

Figure S4. 1D TOCSY spectrum of **1** in $CDCl_3$ at 25°C after selective irradiation of D-forosamine H-6′ at a 120 ms mixing time.

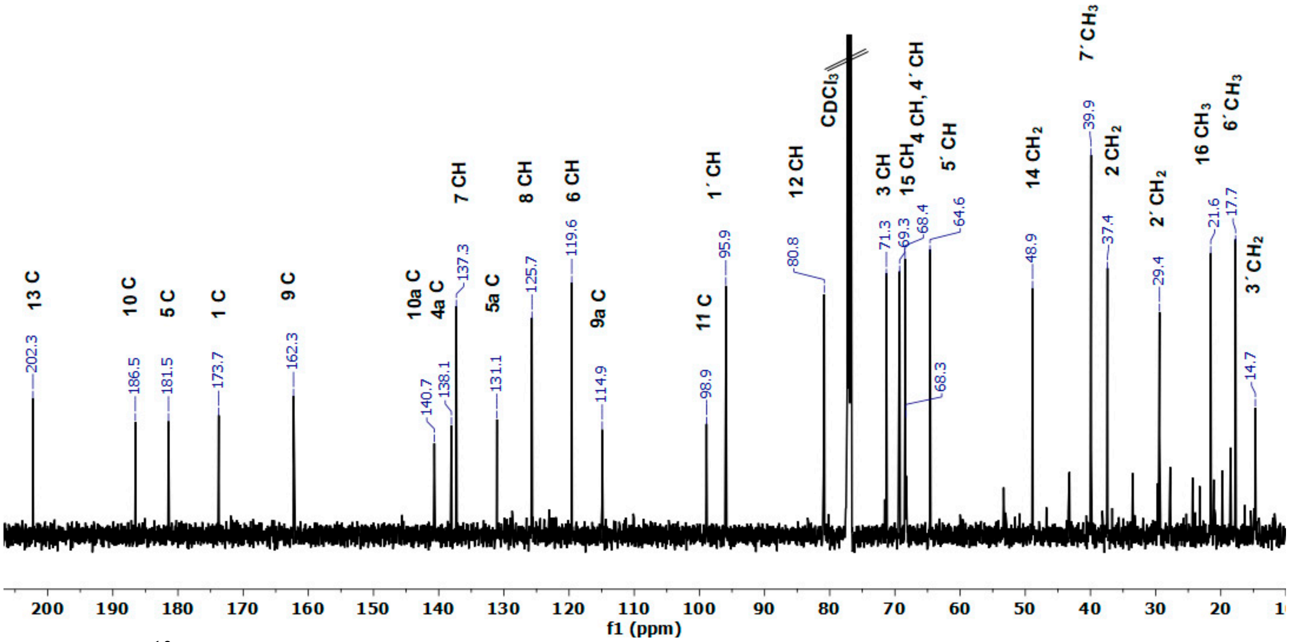

Figure S5. ^{13}C NMR spectrum of **1** in $CDCl_3$ at 25°C.

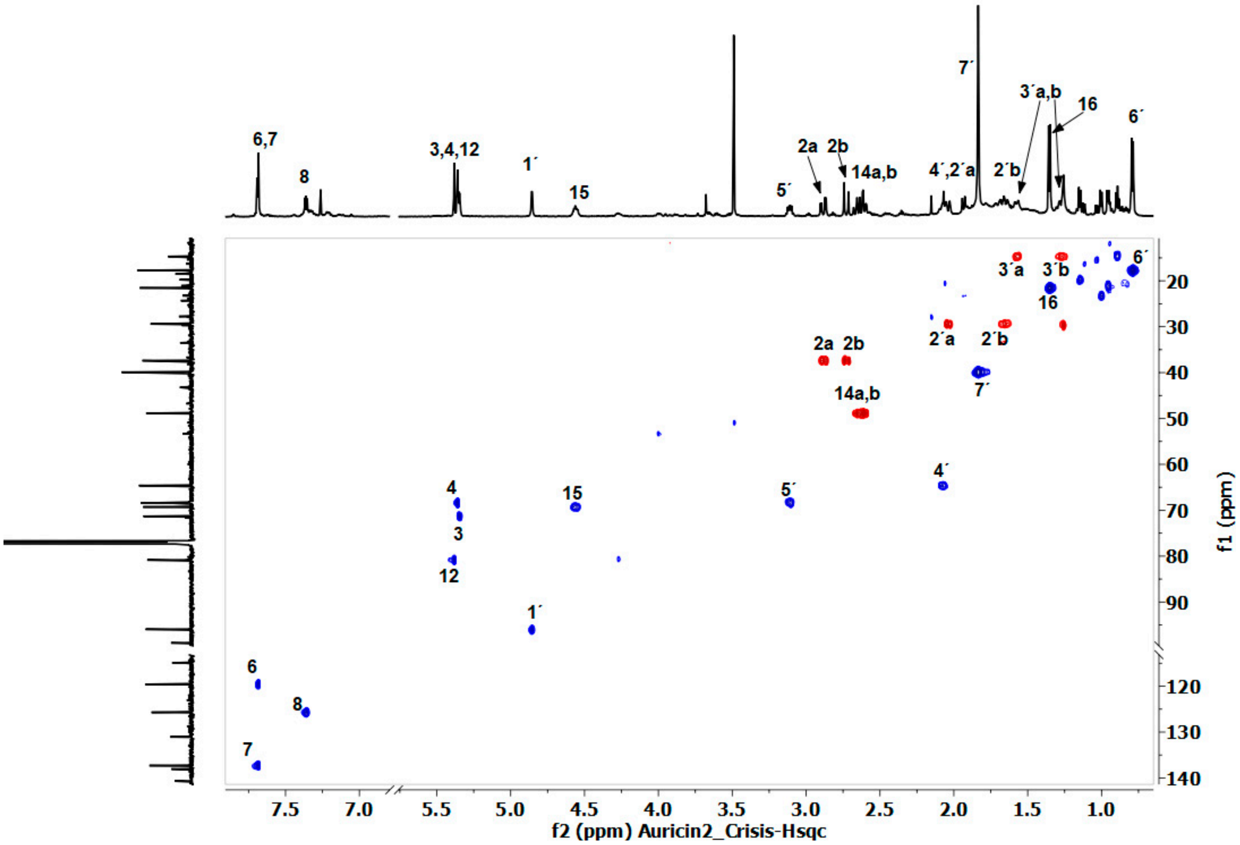

Figure S6. ^{1}H-^{13}C hetero-correlated HSQC spectrum of **1** in $CDCl_3$ at 25°C.

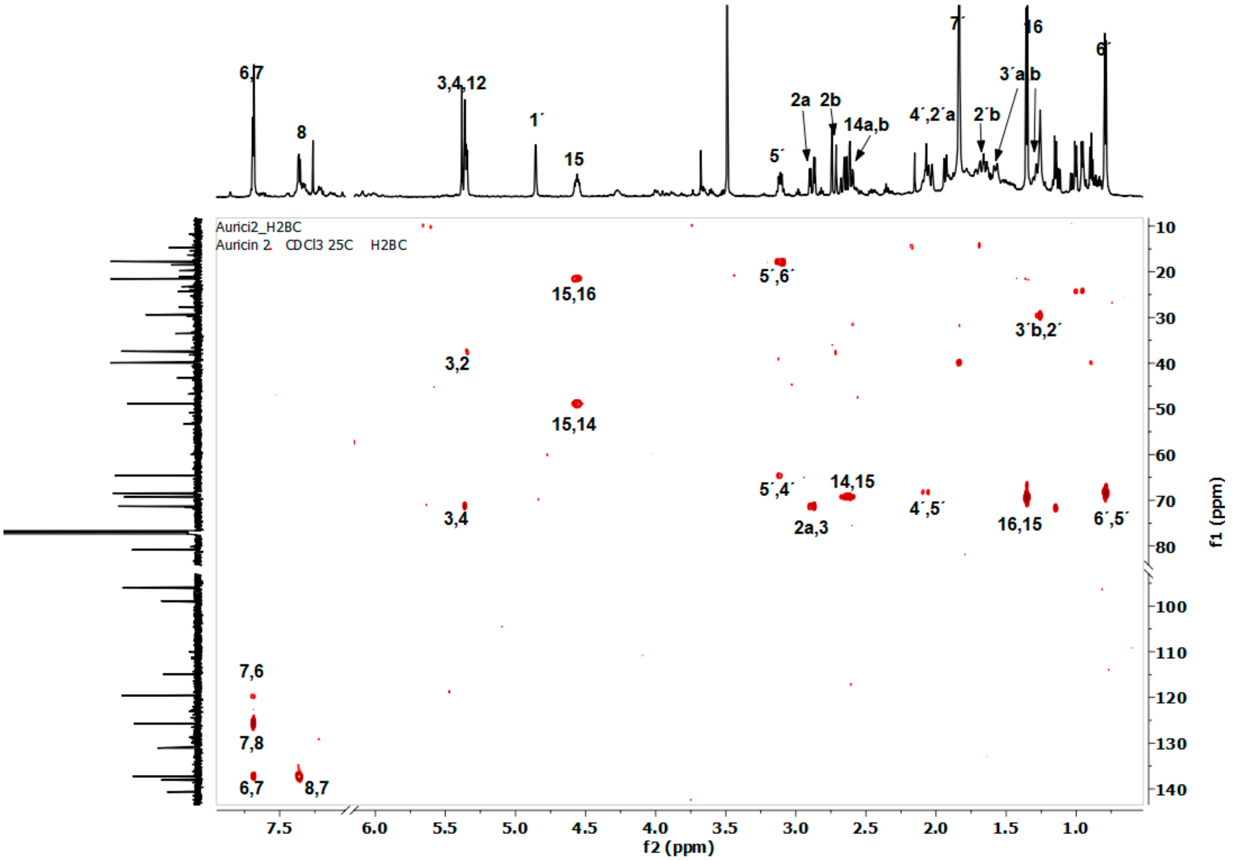

Figure S7. ^{1}H-^{13}C two bond heterocorrelated H2BC spectrum of **1** in $CDCl_3$ at 25°C.

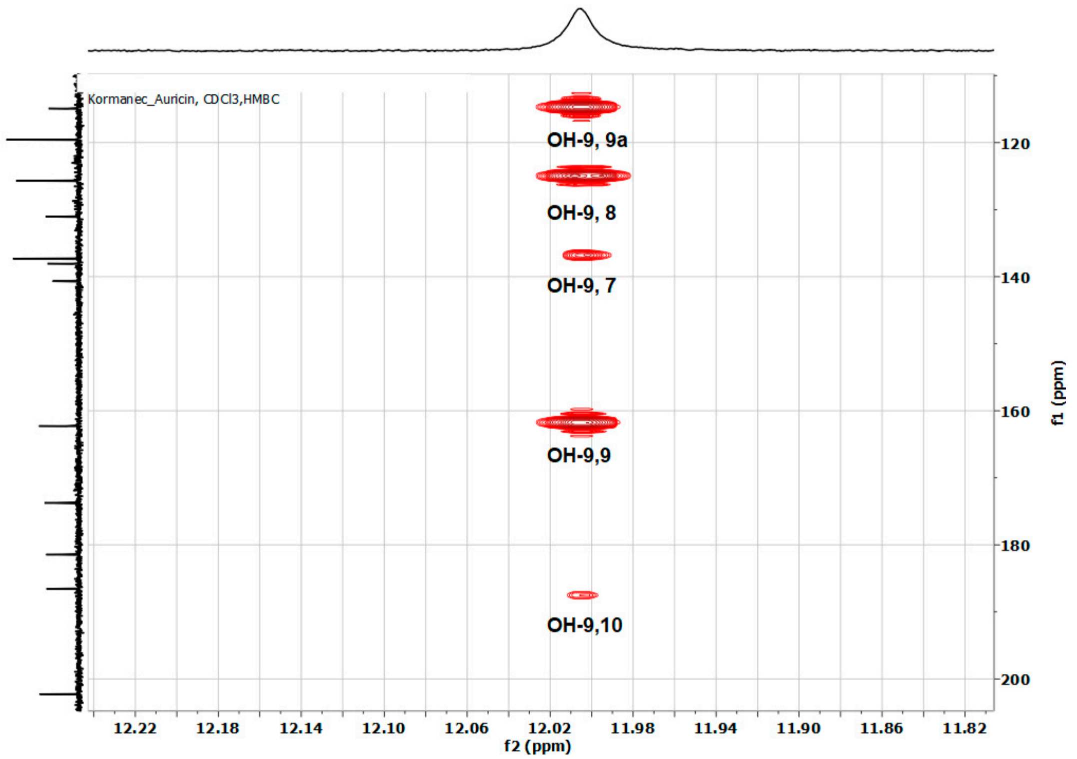

Figure S8. A selected detailed region of the ^{1}H-^{13}C multiple bond correlation HMBC spectrum of **1** in $CDCl_3$ at 25°C.

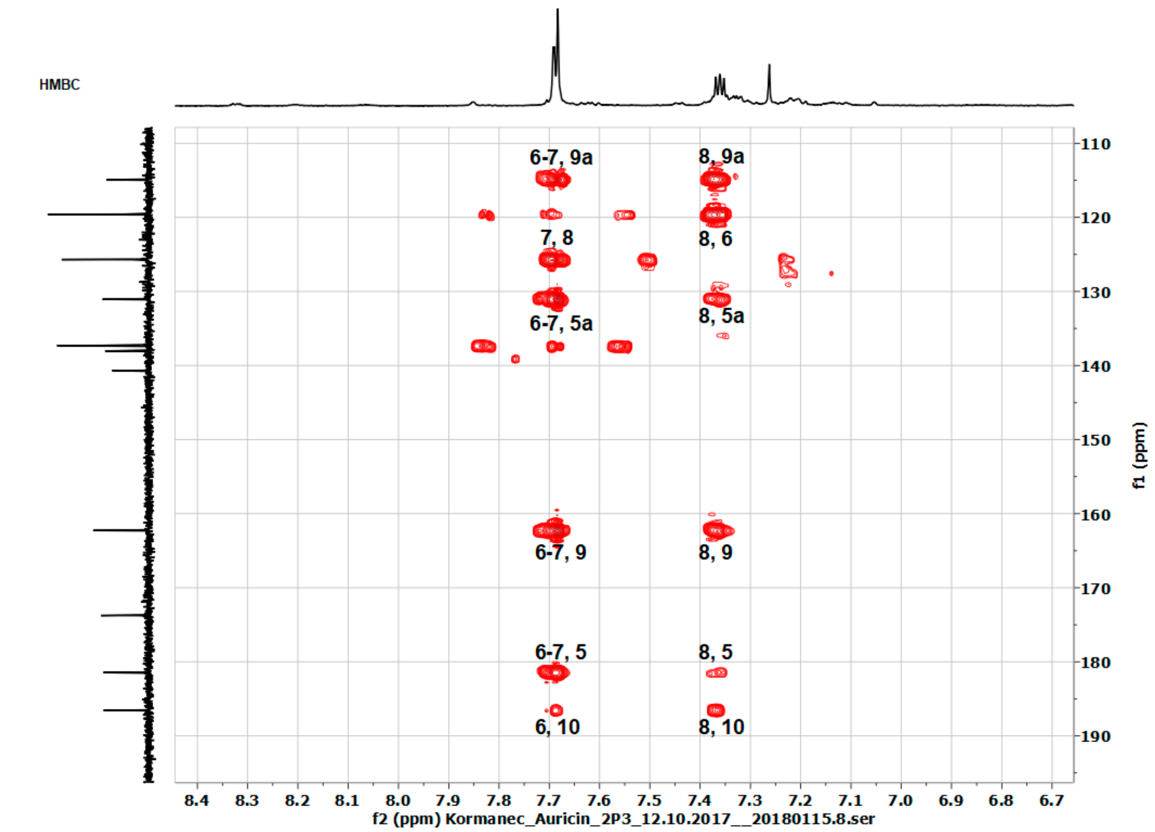

Figure S9. A selected detailed region of the ^{1}H-^{13}C multiple bond correlation HMBC spectrum of **1** in $CDCl_3$ at 25°C.

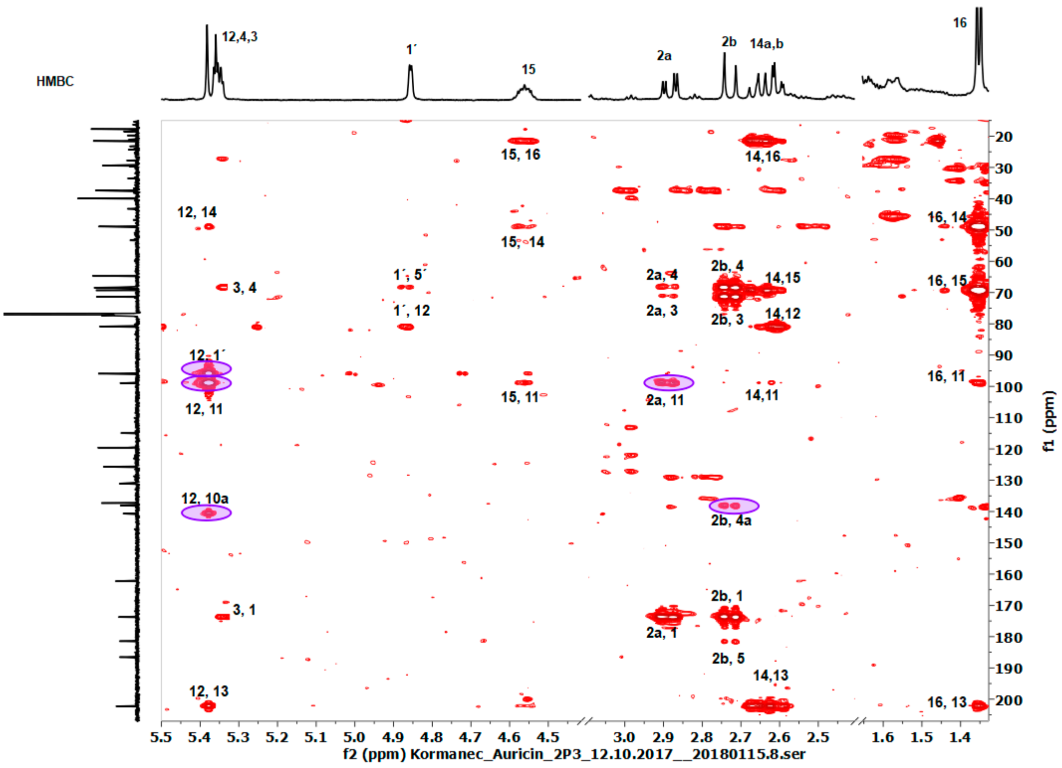

Figure S10. A selected detailed region of the ^{1}H-^{13}C multiple bond correlation HMBC spectrum of **1** (in $CDCl_3$ at 25°C), optimised for a long range coupling constant $^{n}J_{H,C}$ 10Hz. Signals marked with ovals are the most important for the aglycone structure determination.

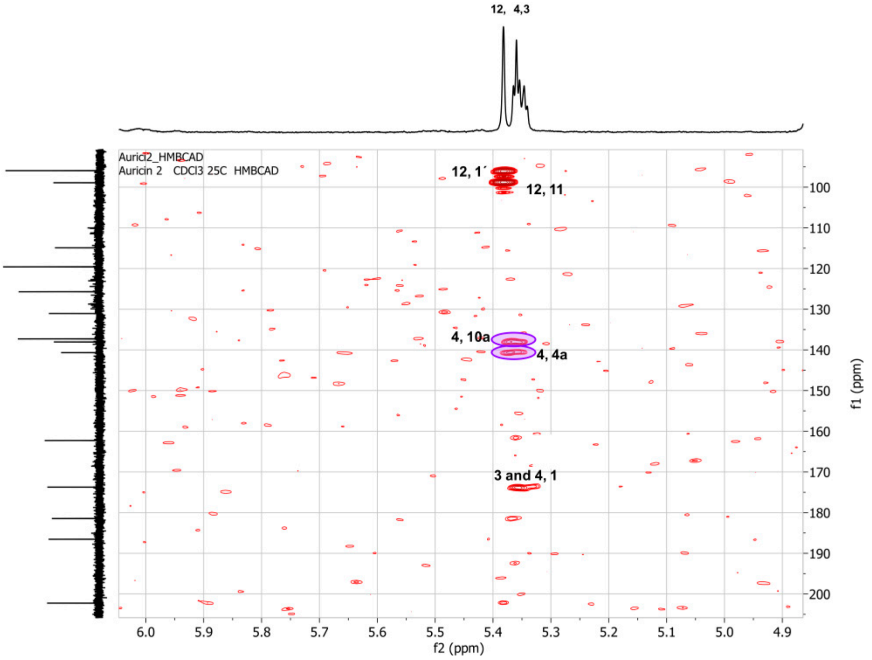

Figure S11. A selected detailed region of the ^{1}H-^{13}C multiple bond correlation HMBC spectrum of **1** (in $CDCl_3$ at 25°C) optimised for a long range coupling constant $^{n}J_{H,C}$ 8Hz. Signals marked with ovals are the most important for the aglycone structure determination.

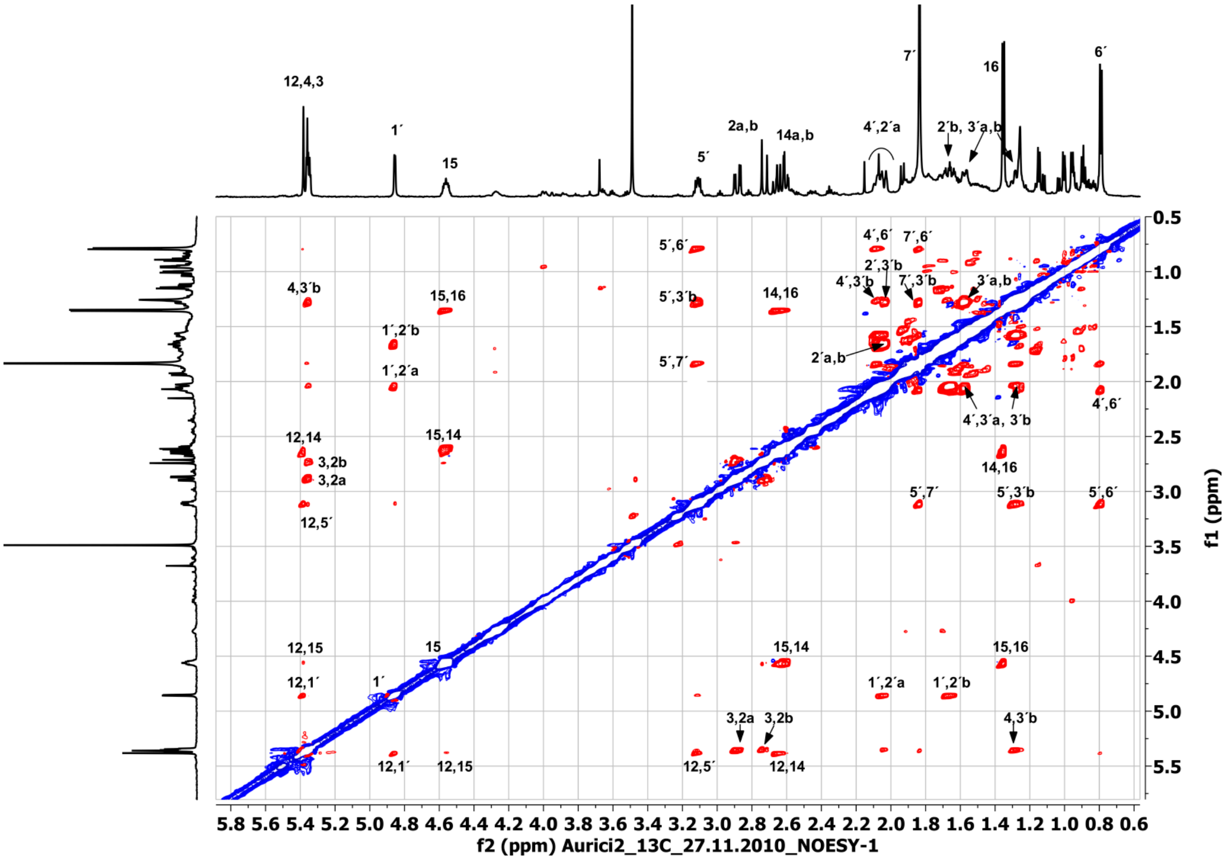

Figure S12. NOESY spectrum of **1** in $CDCl_3$ at 25°C, (mixing time 300ms).

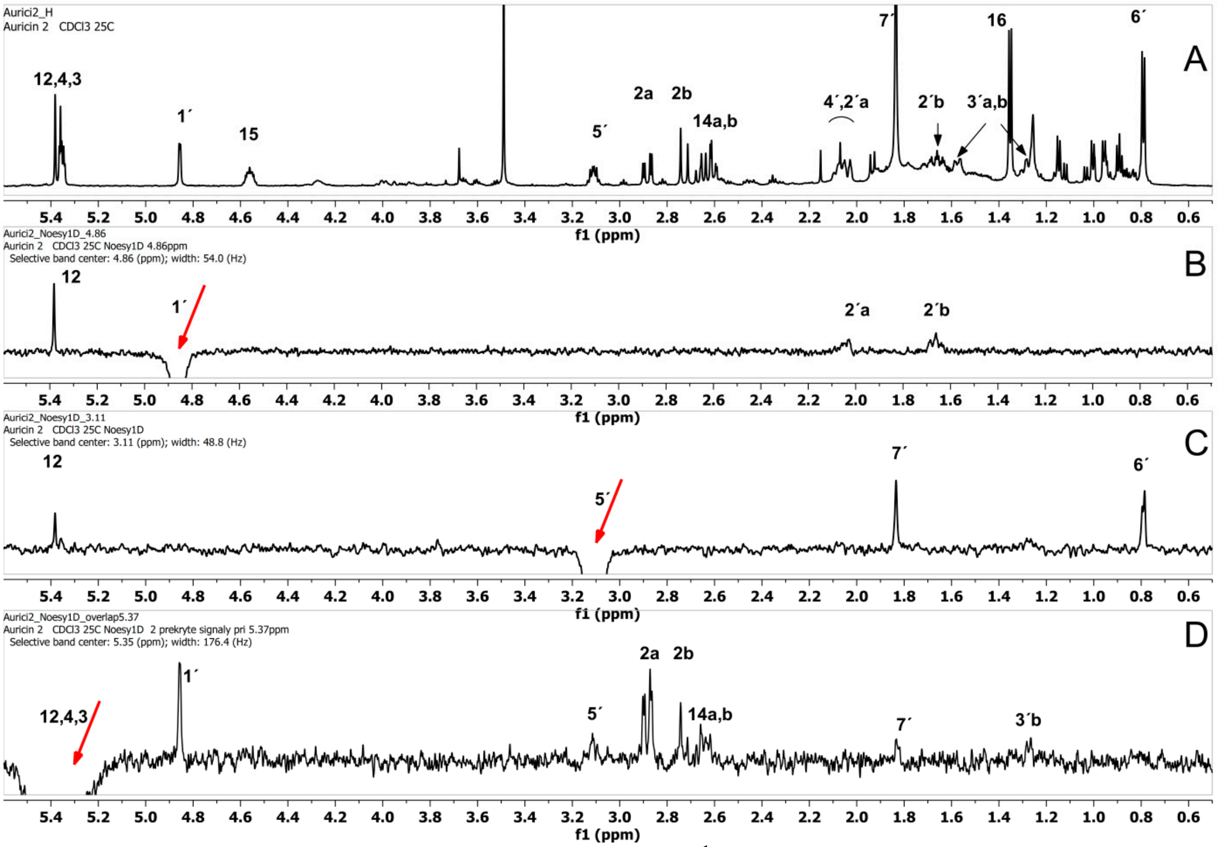

Figure S13. NMR spectra of **1** in $CDCl_3$ at 25°C. A – ^{1}H NMR spectrum of **2**, B and C - 1D NOESY spectrum of **1** after selective irradiation of the anomeric signal H-1´ and the H-5´ signal, respectively, both issued from D-forosamine molecule (mixing time 250ms).

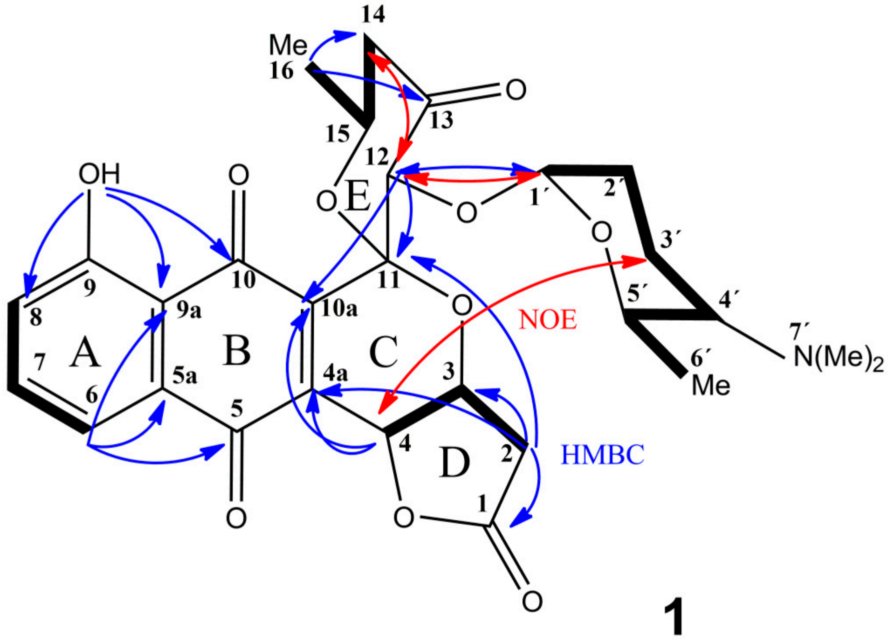

1

Figure S14. The most important correlations of **1**: COSY (bold lines), selected HMBC (blue arrows) and selected NOESY (red arrows).

Table S1 ^{1}H and ^{13}C NMR data (600MHz, $CDCl_3$, 25°C) for auricin (**1**).

Pos.	δC type	δH (ppm) J (Hz)	COSY	ROESY/NOESY[/1]	H2BC[/2]	HMBC[/3] S	M	W	VW
1	173.75, CO								
2	37.39, CH_2	(a) 2.884 (5.2,18.1,1H,dd)<br>(b) 2.727 (18.1,1H, d)	H3		C3	C1<br>C1,C3,C4	C12	C3,C4<br>C4a	 C5
3	71.31, CH	5.351[/4] (1H, dd)	H2, H4[/4]	H2a,b	C2,C4		C1 ,C4		
4	68.45, CH	5.360[/4] (1H, d)	H3[/4]	H3´b	C3		C4a,C10a		C5
4a	138.05, qC								
5	181.45, qC								
5a	131.06, qC								
6	119.59, CH	7.692[/4] (1H)			C7	C8	C5a,C9a	C5,C9	
7	137.29, CH	7.683[/4] (1H)			C6, C8	C5a,C9a,C9	C8,C5		C10
8	125.70, CH	7.361 (4.6, 5.2,1H,dd)			C7	C5a,C6,C9a,C9	C10		C5
9	162.27, qC								
9a	114.92, qC								
10	186.55, qC								
10a	140.68, qC								
11	98.91, qC								
12	80.85, CH	5.382 (1H,s)		H1´, H5´, H14, H15		C1´,C12	C10a,C13		C14
13	202.26, CO								
14	48.88, CH_2	(a) 2.645 (10.7,13.9,1H,dd)<br>(b) 2.604 (3.3, 13.9,1H,dd)	H15	H16<br>H15	C15	C13,C15,C16<br>C11,C13		C11<br>C15	C12<br>C12,C16
15	69.28, CH	4.562 (3.3, 6.4,10.5 1H,m)	H14, H15	H14b, H16	C14, C16	C12,C14,C16			
16	21.55, CH_3	1.353 (6.4,3H, d)	H15	H14a, H15	C15	C14,C15	C13	C12	
OH-9	-	12.00 (1H, s)				C8,C9,C9a		C7	C10
1´	95.94, CH	4.854 (3.0,1H, d)	H2´a,b	H2´a,b; H12	C2´		C11		C5´
2´	29.40, CH_2	(a) 2.041 (1H, m)<br>(b) 1.661 (1H, m)	H1´;H3´a,b	H1´, H3´a,b<br>H1´,	[/n]	[/n]			
3´	14.70, CH_2	(a) 1.576 (1H, m)<br>(b) 1.274 (1H, m)	H2´a,b; H4´	H2´a,b; H4´<br>H2´a, H4´, H5´, H7´	[/n]	[/n]			
4´	64.62, CH	2.08 (1H, m)	H5´	H3´a,b; H5´, H6´	[/n]	[/n]			
5´	68.30, CH	3.111 (1H, m)	H4´, H6´	H3´b, H6´, H7´	C6´	[/n]			
6´	17.74, CH_3	0.792 (6.2, 3H, d)	H5´	H4´, H5´, H7´	C5´	[/n]			
7´	39.91, CH_3	1.835 (6H, s)		H3´b, H5´,H6´	[/n]	[/n]			

[/1] – H-H interaction via dipolar couplings (through space), [/2] – double bond H-C-C interaction through $^{2}J_{H,C}$ scalar couplings, [/3] – long range H-C interaction via $^{n}J_{H,C}$ scalar couplings (through bond); types qC, CH, CH_2, CH_3 mean quaternary, tertiary, secondary and primary carbons, respectively; s - singlet, d- doublet, dd – doublet of doublet, t – triplet, m – multiplet; [/4] – overlapped, [/n] – not assigned. In HMBC column: cross peaks show C-H long range interactions with corresponding proton in the same line; intensity of cross peak signals S -strong, M – medium, W – weak, VW - very weak. (a),(b) – resolved geminal protons of CH_2 group.

Table S2. Comparison of ^{1}H and ^{13}C NMR data for auricin (**1**) (600 MHz, $CDCl_3$, 25°C) with data of equivalent positions in 3´-O-α-D-forosaminyl-(+)-griseusin A (**2**) [15] and 4´-dehydro-deacetylgriseusin A (**3**) [17].

Auricin (**2**) (600 MHz, $CDCl_3$)			3'-O-α-D-forosaminyl-(+)-griseusin A (**2**) (500 MHz, $CDCl_3$)			4´-dehydro-deacetylgriseusin A (**3**) (500 MHz, $CDCl_3$)		
Pos.	δ_C, type	δ_H (J in Hz)	Pos.	δ_C, type	δ_H (J in Hz)	Pos.	δ_C, type	δ_H (J in Hz)
1	173.75, CO	-	2	173.8, CO		12	173.1, CO	
2	37.39, CH_2	2.884, dd (5.2, 18.1)<br>2.727, d (18.1)	3	37.1, CH_2	2.98, dd (4.6, 17.1)<br>2.71, d (17.1)	11	36.0, CH_2	2.94, dd (4.9, 17.8)<br>2.67, d (17.8)
3	71.31, CH	5.351, dd[b]	3a	65.7, CH	4.72, dd (2.4, 4.3)	10	66.7, CH	4.68, dd (3.0, 4.8)
4	68.45, CH	5.36, d[b]	11b	68.2, CH	5.26, d (2.7)	9	68.1, CH	5.27, d (3.0)
4a	138.05, qC	-	11a	138.2, qC		8a	138.5, qC	
5	181.45, qC	-	11	181.8, qC		8	181.7, qC	
5a	131.06, qC	-	10a	131, qC		7a	131.2, qC	
6	119.59, CH	7.692[b]	10	119.8, CH	7.68, dd (1.8, 7.9)	7	119.7, CH	7.65, m
7	137.29, CH	7.683[b]	9	137.1, CH	7.65, t (7.9)	6	137.2, CH	7.65, m
8	125.7, CH	7.361, dd (4.6, 5.2)	8	125.6, CH	7.32, dd (1.8, 7.9)	5	125.5, CH	7.30, m
9	162.27, qC	-	7	162.3, qC		4	162.2, qC	
9a	114.92, qC	-	6a	115.3, qC		3a	115.2, qC	
10	186.55, qC	-	6	187.2, qC		3	187.3, qC	
10a	140.68, qC	-	5a	143.7, qC		2a	140.5, qC	
11	98.91, qC	-	5, 2'	96.1, qC		2	99.4, qC	
12	80.85, CH	5.382, s	3'	69.7, CH	4.90, d (3.6)	3'	75.9, CH	5.47, d (8.6)
13	202.26, CO	-	4'	64.6, CH	5.59, q (3.6)	4´	203.0, CO	
			4' (-OAc)	21.1, CH_3	2.08, s			
			4' (-OAc)	170.4, CO				
14	48.88, CH_2	2.645, dd (10.7, 13.9)<br>2.604, dd (3.3, 13.9)	5'	36.0, CH_2	1.91, m	5´	47.6, CH_2	2.57, m
15	69.28, CH	4.562, m (3.3, 6.4, 10.5)	6'	63.0, CH	4.37, m	6´	69.5, CH	4.21, m
16	21.55, CH_3	1.353, d (6.4)	7'	20.6, CH_3	1.24, d (6.4)	7´	21.4, CH_3	1.39, d (6.2)
OH-9	-	12.00, s				OH-4		11.9, s
1´	95.94, CH	4.854, d (3.0)	1"	93.7, CH	4.82, s			
2´	29.4, CH_2	2.041, 1.661, m	2"	29.7, CH_2	1.47, 1.61, m			
3´	14.7, CH_2	1.576, 1.274, m	3"	13.6, CH_2	1.47, m			
4´	64.62, CH	2.08, m	4"	65.7, CH	1.95, m			
5´	68.3, CH	3.111, m	5"	68.0, CH	3.19, d (6.4, 9.8)			
6´	17.74, CH_3	0.792, d (6.2)	6"	18.2, CH_3	0.58, d (6.4)			
7´	39.91, CH_3	1.835, s	4"-N-$(CH_3)_2$	40.3, CH_3	1.92, s			

Types qC, CH, CH_2, CH_3 mean quaternary, tertiary, secondary and primary carbons, respectively. CO – carbonyl, s – singlet, d – doublet, t – triplet, dd – doublet of doublet, [b] – overlapped. Highlighted yellow positions show significantly different values.

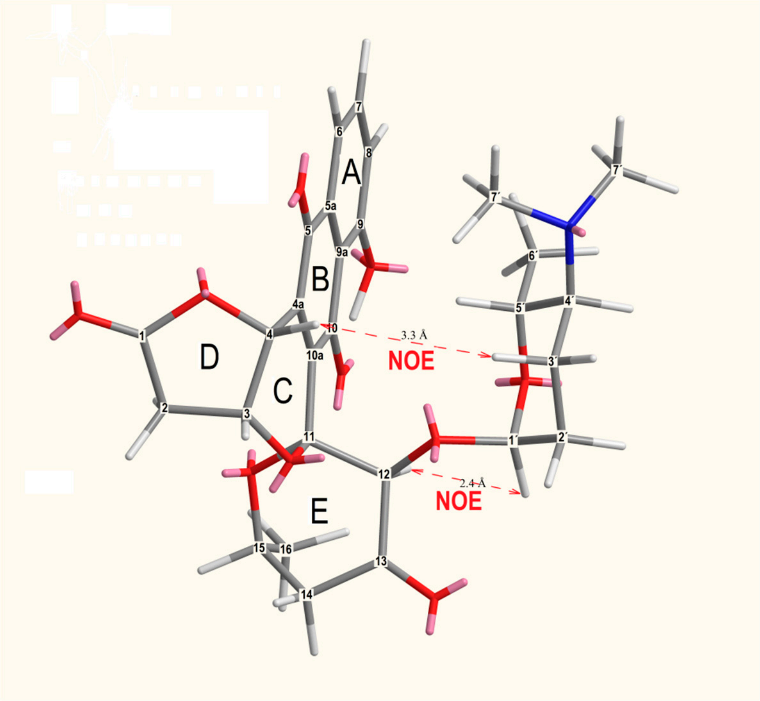

Figure S15. Three dimensional structure of **1**. It has resulted from MM2 force field for minimum energy calculation in Chem3D Pro program.

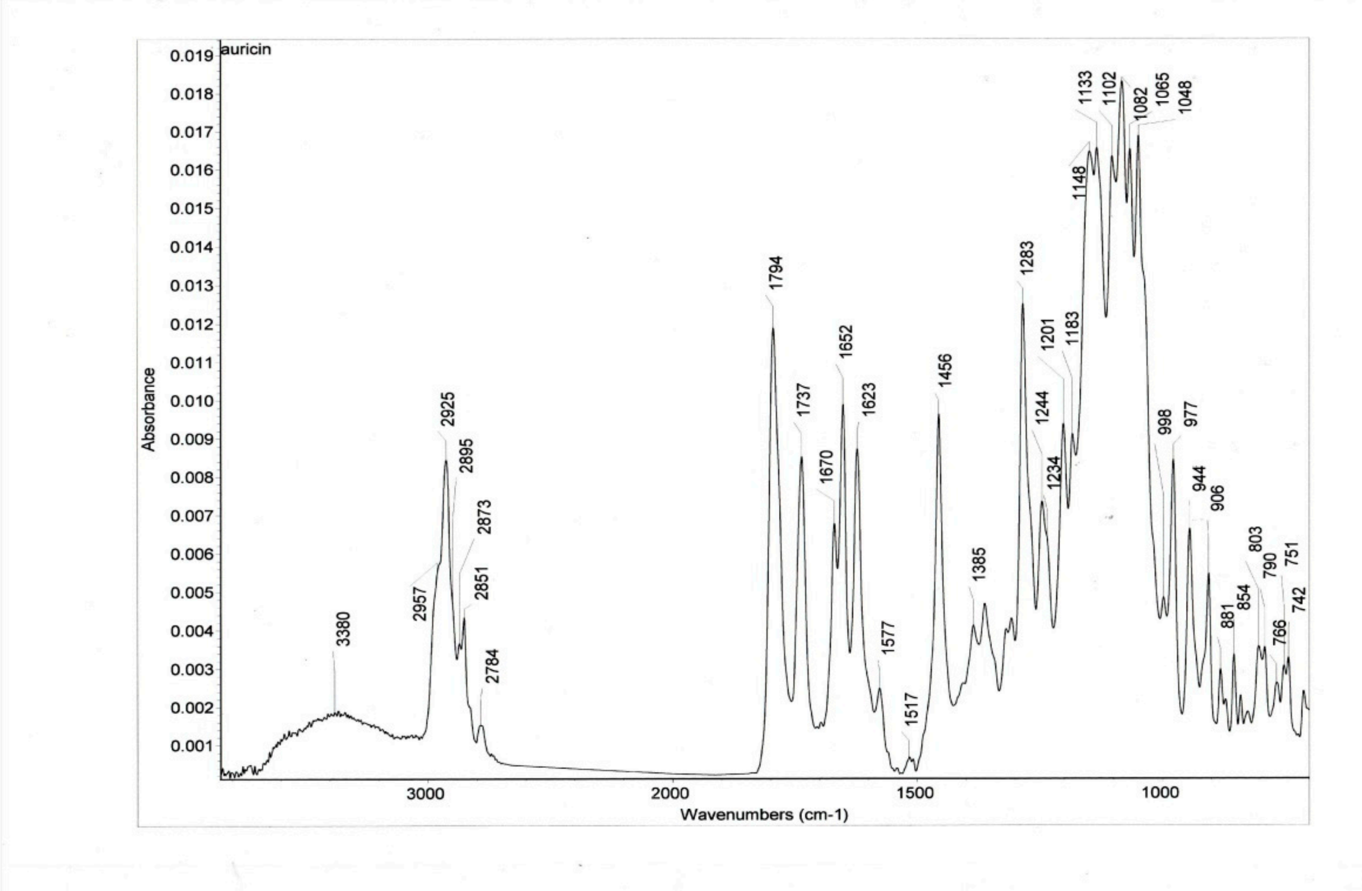

Figure S16. FT-IR ATR Infrared spectrum of **1.**

Table S3 Characteristic FTIR bands identified in the spectrum of **1**. The signals were assigned based on Miller and Willis, 1969.

wavenumber (cm^{-1})	assignment
2957	ν_{as} (C-H) of CH_3
2925	ν_{as} (C-H) of CH_2
2895	ν (C-H) of CH
2873	ν_{s} (C-H) of CH_3
2851	ν_{as} (C-H) of CH_2
2784	ν (O-H) chelation of OH-9 with C-10 carbonyl
1794	ν (C=O) of ketone C-13
1737	ν (C=O) of ester (ring A)
1670	ν (C=O) of ketone C-5
1652	ν (C=O) of ketone C-10
1623	ν (C=C) arom.
1577	ν (C=C) arom.
1517	ν (C=C) arom.
1456	δ (C-H)
1385	δ (C-H)
1283	δ (O-H) phenolic
1244	ν (C-O) of ester (ring A)
1234	ν (C-N) of terc.amine
1201	ν (C-O-C) of cyclic ether (ring B) ^{3}C-O-^{12}C
1183	ν (C-O-C) of cyclic ether (ring E) ^{15}C-O-^{12}C
1148	ν (C-O-C) of glycosidic ^{11}C-O-$^{1'}C$
1133	ν (C-N) of terc.amine
1082	ν (C-O-C) of ether $^{1'}C$-O-$^{5'}C$

ν- stretching vibrations, δ - deformation vibrations, ^{n}C stands for carbon in position n

References

Miller, R. G. J. & Willis, H. A. *Irscot: Infrared structural correlation tables and data cards.* Irscot System – Tables 1-9. Heyden & Son Ltd., London, 1969

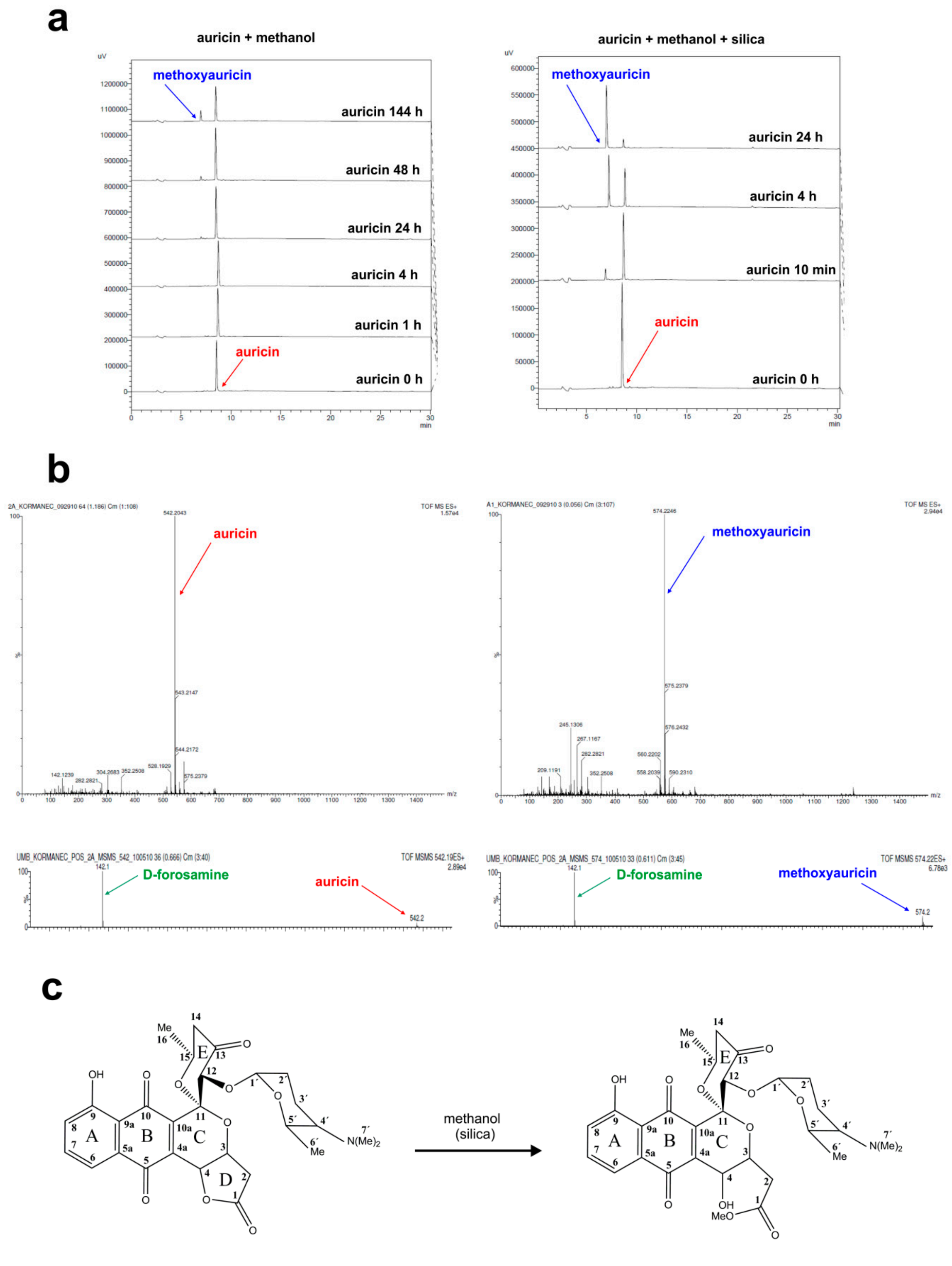

auricin (**1**) (mass=541.2)

methoxyauricin (mass=573.2)

Fig. S17. (a) HPLC analysis of conversion of compound **1** to methoxyauricin in the presence of methanol (methanolysis), and catalysed by silica. 24 µg of purified compound **1** was dissolved in 120 µl methanol and incubated in room T with and without silica. At the time point indicated, 10 µl of the sample was analysed by analytical HPLC as described previously [7,9]. Traces show the elution of metabolites monitored at 245 nm (*y*-axis) versus time (*x*-axis). **(b)** High resolution ESI MS spectra of compound **1** and methoxyauricin and ESI MS/MS fragmentation of compound **1** and methoxyauricin to give the D-forosamine *m/z* = 142.2 {M+H]$^{+}$ signal. **(c)** Scheme of conversion of compound **1** to methoxyauricin.

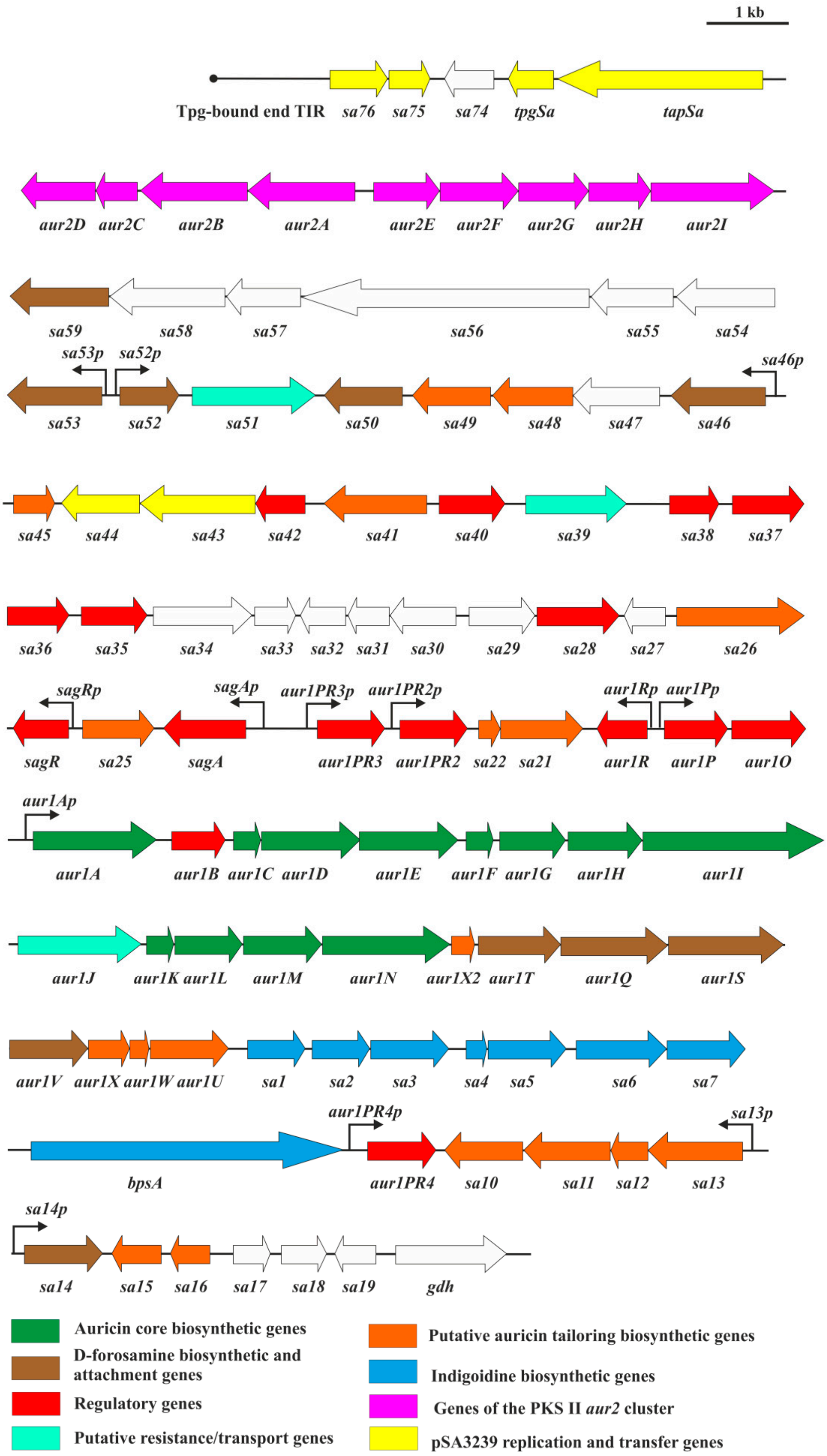

Figure S18. Genetic organisation of the auricin (**1**) *aur1* cluster and its adjacent regions. Each thick arrow denotes the direction of expression and the size of the gene. Details of individual genes and their products are provides in Genbank Acc. No. KJ396772. Bent arrows indicate the position and direction of promoters regulated by auricin-specific regulators [5,8,9].

```
Gra-ORF1        -------------------------------------------VTRRVVITGVGVRAPGG
Qin-ORF19       -------------------------------------------MTRRVVITGIGVRAPGG
Med-ORF1        -------------------------------------------MTRRVVVTGLGVRAPGG
ActI-ORF1       VPLDAAPVDPASRGPVSAFEPPSSHGADDDDDHRTNASKELFGLKRRVVITGVGVRAPGG
AlnL            -------------------------------------------VNRSVAITGIGVVAPGG
FrnL            -------------------------------------------VNRQVAVTGIGVVAPGG
AlpA            ------------------------------------------LSRRRVVITGIEVIAPGG
JadA            ------------------------------------------VTARRVVITGIEVLAPGG
LanA            -------------------------------------------MGRRVVVTGIGVLAPGG
PgaA            -------------------------------------------VSRRVVITGVGVLAPGG
UrdA            ---------------------------------------VSGAHSRRVVITGIGVTAPGG
SimA1           -------------------------------------------VRRRVVITGVGVMAPGG
Aur1D           -------------------------------------------MTRRVVITGIGVLAPGG
Gris-ORF1       -------------------------------------------VERRAVITGIGVCAPGG
ElmK            ---------------------------------------MTG---RQVVITGIGVRAPGG
TcmK            ---------------------------------------MTRHAEKRVVITGIGVRAPGG
PdmA            ------------------------------------VSRPQGGGPRRVAITGMGVVAPGG
MtmP            -------------------------------------------MNRRVVITGIGVVAPGA
Dau-ORFA        -------------------------------------------VNRRVVITGMGVVAPGA
DpsA            -------------------------------------------VNRRIVITGIGVVAPGA
OxyA            --------------------------------------MSKIHDARRVVITGIGVVAPGD
                                                             :  .:**: * ***

Gra-ORF1        SGTKEFWDLLTAGRTATRPISFFDASPFRSRIAGEIDFDAVAEGFSPREVRRMDRATQFA
Qin-ORF19       SGTKEFWDLLTAGRTATRRISFFDAAAFRSQVAAEADFFPEAEGFSPREIRRMDRATQFA
Med-ORF1        SGARQFWDLLSSGRTATRSITSFDASACRSQVAGEIDFDPVAEGLSPREIRRMDRAAQFA
ActI-ORF1       NGTRQFWELLTSGRTATRRISFFDPSPYRSQVAAEADFDPVAEGFGPRELDRMDRASQFA
AlnL            VGKKAFWDLLVSGRTATRTISFFDPSRFRSQVAAEVDFDPQRSGLSPREARRLDRAAQFA
FrnL            IGRKPYWEQLTSGRTATRAISFFDASPFRSRIAAEVDFDPAAAGLSPREVRRMDRAAQFA
AlpA            VGRENFWNLLSSGRTATRGITFFDPAPFRSRVAAEADFDPFAHGLSPQEVRRLDRAAQFA
JadA            TGSKAFWNLLSEGRTATRGITFFDPTPFRSRVAAEIDFDPEAHGLSPQEIRRMDRAAQFA
LanA            IGAENFWSLLSEGRTATRGITFFDPSSFRSQIAAEADFDAERSGLSPQEIRRMDRAAQFG
PgaA            IGAKNFWSLLSEGRTATRGITFFDPSSFRSRVAAEADFDPEMHGLTPQEIRRMDRAAQFA
UrdA            VGSKNFWSLLSDGRTATRRISFFDPSPFRSQVAAEADFDAELLGLSPQEIRRMDRAAQFA
SimA1           IGVKNFWSLLSEGRTATRGITFFDPAPFRSRVAAEIDFFPEKHGLGPQKIRRMDRAAQLA
Aur1D           IGTTAFWNLLSSGRTATRGITLFDPAPFRSRVAAEVDFHPERHGLTSLEVRRMDRAAQFG
Gris-ORF1       TGVDSFWELMCDGRTATRGITLFDPSPYRSRVAAEIDFDPRAHGLTPQEIRRMDRAAQFG
ElmK            TGVKAFWDLLSSGRTATRRISSFDPSPYRSQIAAEIDFDPLGEGFTDRQIARWDRAVLLA
TcmK            AGTAAFWDLLTAGRTATRTISLFDAAPYRSRIAGEIDFDPIGEGLSPRQASTYDRATQLA
PdmA            SGRKAFWNLLTDGRTATRKISLFDPAGFRSRIAAECDFDPAAEGLTPREVRRMDRAAQLA
MtmP            VGVEAFWEQLTAGRTATRTISLFDASAFRSRIAAEVDFDAARHGFGPAEAERLDRATQFA
Dau-ORFA        IGIKSFWELLLSGTTATRAITTFDATPFRSRIAAECDFDPVAAGLSAEQARRLDRAGQFA
DpsA            VGTKPFWELLLSGTTATRAISTFDATPFRSRIAAECDFDPVAAGLSAEQARRLDRAGQFA
OxyA            VGTKPFWEMLTAGRTATRPISSFDASPFRSQVAAECDFDPAAEGLSQRQVRAWDRTMQFA
                 *   :*. :  * **** *: **.:  **::*.* ** .   *:   :    **:  :.

Gra-ORF1        VACTRDALADSGLDTGALDPSRIGVALGSAVASATSLENEYLVMSDSGREWLVDPAHLSP
Qin-ORF19       VACTREAVADSGLEFAGVDPHRVGVSLGSAVASATSLENEYLVMADKGREWLVDPDYLSP
Med-ORF1        VVCSREAVADSGLSFEGVRPERIGVSVGSAVAAAMSLEKEYRVLSDQGREWEVDPTYLTP
ActI-ORF1       VACAREAFAASGLDPDTLDPARVGVSLGSAVAAATSLEREYLLLSDSGRDWEVDAAWLSR
AlnL            VVSARECMADSGLEFVELDPHRTGVSVGSAVGGTTGLEREYLVLSDSGRLWEVDSDYVSP
FrnL            VVSARESLADSGLDVADLDPHRIGVSIGSAVGGTTSLEREYLALSDSGRQWELDLSYLSP
AlpA            VVASRGAVADSGLDVAALDPHRVGVTVGSAVGATMGLDEEYRVVSDGGRLEAVDHTYAVP
JadA            VVAAR-AVADSGIDLAAHDPYRVGVTVGSAVGATMGLDEEYRVVSDGGRLDLVDHAYAVP
LanA            VVTAREALADSGLDQAGLDPYRTGVTIGSAVGATMGLDEEYRVVSDGGRLDLVDHRYTPQ
PgaA            VVTAREALADSGLDLAGFDPHRTGVTIGSAVGATTGLDDEYRVVSDGGRLDLVDHTYAPP
UrdA            VVTAREAVADSGLEFASLDPHRTGVTVGSAVGATMGLDQEYRTVSDSGRLDLVDHEYAVP
SimA1           VIATREAVADSGLDLDATDPHRRGVTVGSAVGATTGLDQEYRVVSDDGRLGLVDHRYAPP
Aur1D           VVATREALADSGLDLAAFDPYRIGIAMGTGVGAISGLDTAYRVASDEGRIAQVDHMYAPG
Gris-ORF1       VVTAREAFADSGLDGAALDPFRTGVSLGTAIGAAGGLDAEYRVVSDDGRLELVDHRYATP
ElmK            VAAAREALAHSGLAPGALRPETVGVSVGSAVGCTTSLDTEYARVSHGGADWLVDHTLAVQ
TcmK            VVCAREALKDSGLDPAAVNPERIGVSIGTAVGCTTGLDREYARVSEGGSRWLVDHTLAVE
PdmA            VVSAREALADSGLVAGEGDPARFAVSLGSAVGCTMGLEDEYVVVSDQGRDWLVDHSYGVP
MtmP            LVSAREAVADAGLDG-KTDPSRTGVALGSAVGCTTGLDTQYNVVSEGGSDWYVDHTRAVD
Dau-ORFA        LVAGQEALTDSGLRIGEDSAHRVGVCVGTAVGCTQKLESEYVALSAGGANWVVDPHRGAP
DpsA            LVAGQEALADSGLRIDEDSAHRVGVCVGTAVGCTQKLESEYVALSAGGAHWVVDPGRGSP
OxyA            YVAAREALADSGV-TGEADPLRTGVMAGTACGMTMSLDREYAVVSDEGRLWQVDDAHGVP
                    : ..  :*:      .   .:  *:. .    *:  *   :  *    :*
```

```
Gra-ORF1        MMFDYLSPGVMPAEVAWAAGAEGPVTMVSDGCTSGLDSVGYAV-QGTREGSADVVVAGAA
Qin-ORF19       HMFDYLSPGVMPAEVAWAAGAEGPVTMVSDGCTSGLDAVGYGV-QLIREGSADVMIAGAS
Med-ORF1        HMFDHMVPSVLGAEVAWTVGAEGPVTVVSDGCTSGLDSVGYAC-RLIQEGSVDVMLAGAT
ActI-ORF1       HMFDYLVPSVMPAEVAWAVGAEGPVTMVSTGCTSGLDSVGNAV-RAIEEGSADVMFAGAA
AlnL            HLFDAFVPSSLAAEVAWTVGAEGPATVVSTGCTSGLDSVGYAR-DLIAEGTVDVMIAGAA
FrnL            HLYDAFTPSSLAREVAGVIGAEGPAAVVSTGCTSGIDSLGHAR-DLIAEGSADVVLAGGT
AlpA            HLYDYMVPSSFAAEVAWAVGAEGPSTVVSTGCTSGIDSVGYAV-ELVREGSADVVIAGSS
JadA            HLYDYMVPSSFSAEVAWAVGAEGPNTVVSTGCTSGLDSVGYARGELIREGSADVMIAGSS
LanA            HLYNHFVPSSFSAEVAWAVGAEGPNTVVSTGCTSGIDSVGQAV-ELIREGSVDVMIAGAT
PgaA            HLYNYFVPSSFSAEVAWAVGAEGPATVVSTGCTSGLDSVGHAT-ELIREGTADVMITGAT
UrdA            HLYNYLVPSSFAAEVAWAVGAEGPATVVSTGCTSGLDAVGYAT-ELIREGSADVMVAGAA
SimA1           HLYGHFVPSSFAAEVAWASGSEGPSTVVSTGCTSGLDSLGHAV-ELIREGSADVMIAGAT
Aur1D           HLYDHFVPSSFATEVAWAVGAEGPSTVVSTGCTSGIDSVAHAV-DLIREGSADIMITGAT
Gris-ORF1       HLYDLFVPSSFATEVAWAVGAEGPATVVSTGCTAGIDAVGHAV-EAIRDGSADVMVTGAV
ElmK            QLFDYFVPTSISREVAWEVGAEGPVTLVSTGCTSGLDAVGNGA-SLIRDGNADVVLAGAT
TcmK            QLFDYFVPTSICREVAWEAGAEGPVTVVSTGCTSGLDAVGYGT-ELIRDGRADVVVCGAT
PdmA            HLYRHLVPSSLAAEVAWAGGAEGPVTLISTGCTSGLDAVGHGA-RVIAEGSADVALAGAT
MtmP            HLFDYFVPSSMAAEVAWSIGAQGPVALISTGCTSGLDSLGHAV-ALIREGSADVMVAGAT
Dau-ORFA        ELYDYFVPSSLAAEVAWLAGAEGPVNIVSAGCTSGIDSIGYAC-ELIREGTVDVMLAGGV
DpsA            ELYDYFVPSSLAAEVAWLAGAEGPVNIVSAGCTSGIDSIGYAC-ELIREGTVDAMVAGGV
OxyA            YLYDYFVPSSMAAEIAWLAEAEGPAGVVSAGCTSGIDVLTHAA-DLVRDGAAEVMVAGAS
                 ::  : *  :  *:*    ::**  ::* ***:*:* :  .      :* .:  . *.

Gra-ORF1        DTPVSPIVVACFDAIKATTPRNDDPAHASRPFDGTRNGFVLAEGAAMFVLEEYEAAQRRG
Qin-ORF19       DTPISPIVLACFDAIKATTTRNDDPEHASRPFDASRNGFVLAEGSAMFVLEEYEAAKARG
Med-ORF1        DTPLTPIVAACFDAIKATTPRNDDAEHASRPFDLSRNGFVLAEGAAMFVLEEYESAVRRG
ActI-ORF1       DTPITPIVVACFDAIRATTARNDDPEHASRPFDGTRDGFVLAEGAAMFVLEDYDSALARG
AlnL            DTPISPIAVSCFDAIKATTPRNDDPEHASRPFDRTRNGFVLAEGAAMFVLEELEHARARG
FrnL            DTPISPIAVACFDAIKATSPSNDDPAHASRPFDRERNGFVLAEGAAVLVLEELGHARARD
AlpA            DAPISPITMACFDAIKATTPRHDEPECASRPFDKTRNGFVLGEGCAFFVLEELDSARKRG
JadA            DAPISPITMACFDAIKATTNRYDDPAHASRPFDGTRNGFVLGEGAAVFVLEELESARARG
LanA            DAPISPITMACFDAIKATTPRNDEPEHASRPFDGTRNGFVLGEGSAVFVLEELTAARARG
PgaA            DAPISPITMACFDAIKATTPRNDDAAHASRPFDGTRNGFVLGEGSAVFVLEELESAKKRG
UrdA            DAPISPITVACFDAIKATTPRNEDPEHASRPFDGTRNGFVLGEGSAVFVLEELDSALRRG
SimA1           DAPISPITMACFDAIKATTPRNDDPAHASRPFDRSRNGFVLGEGHAVLVLEELGAAQRRG
Aur1D           DAPISPLTLACFDAIKATTALNEEPETASRPFDATRKGFVLGEGAAAFVLEELGAARRRG
Gris-ORF1       DAPITPLTMAAFDAIRATTSLNDEADTASRPFDASRKGFVLGEGAAVFVLEEAEHARRRG
ElmK            DAPISPITVACFDAIKATSPNNADPEHASRPFDRRRDGFVLGEGAAMFVLEEKEAALRRG
TcmK            DAPISPITVACFDAIKATSANNDDPAHASRPFDRNRDGFVLGEGSAVFVLEELSAARRRG
PdmA            DAPISPITVACFDAIRATSPNNDDPEHASRPFDRERNGFVLGEGAAVFVLEELEHARRRG
MtmP            EAPISPITVACFDAIKATSPRNDDPATASRPFDRSRNGFVLGEGSAVLILESLENARRRD
Dau-ORFA        DAPIAPITVACFDAIRVTSDHNDTPETLA-PFSRSRNGFVLGEGGAIVVLEEAEAAVRRG
DpsA            DAPIAPITVACFDAIRATSDHNDTPETASRPFSRSRNGFVLGEGGAIVVLEEAEAAVRRG
OxyA            DAAISPITVACFDAIKATTPRNDEPETASRPFDRTRNGFVLGEGAAFFVLEEYAHARRRG
                ::.::*:. :.****:.*:     .   : **.  *.****.** * .:**.   *  *.

Gra-ORF1        AHIYAEVGGYATRSQAYHMTGLKKDGREMAESIRAALDEARLDRTAVDYVNAHGSGTKQN
Qin-ORF19       AQIYAEVTGYATRCNAYHMTGLKKDGREMAEAIRAALDESRVDPTVVDYVNAHGSGTKQN
Med-ORF1        ARIYAEVTGYATRLNAHHMTGLKTDGREMAEAIRVALDESRIDPTAIDYVNAHGSGTKQN
ActI-ORF1       ARIHAEISGYATRCNAYHMTGLKADGREMAETIRVALDESRTDATDIDYINAHGSGTRQN
AlnL            AHVYGVIGGYATRCNAYHMTGLRPDGHEMAEAIRHSLDQARLNPDLVDYVNAHGSGTKQN
FrnL            AHVYALVSGYATRCNAYHMTGLTPHGREMAEAIRHALAESGTDPAAVDYVNAHGSGTKQN
AlpA            AHIYAEIAGYSTRSNAYHMTGLRPDGAEMAEAIDLALAEARLNPQAIDYVNAHGSGTKQN
JadA            AHIYAEIAGYATRSNAYHMTGLRPDGAEMAEAIRVALDEARMNPTEIDYINAHGSGTKQN
LanA            AHIYAEIAGYASRCNAFHMTGLRPDGREMGEAIRVALDEARINPEAIDYINAHGSGTKQN
PgaA            AHIYAEIAGYASRCNAFHMTGLRPDGREMSEAIDVALGEARMNPDRIDYINAHGSGTKQN
UrdA            AHVYAEIAGYATRSNAFHMTGLRPDGREMAEAIRIALDEARLNPEDIDYVNAHGSGTKQN
SimA1           ARIYAEIGGYASRCNAFHMTGLRPDGREMAEAIRVALDEARLNPEDIDYINAHGSGTKQN
Aur1D           AHVYAEIAGYASRSSAFHMTGLRPDGREMAEAITVALDEARLDPGDLDYVNAHGSGTRQN
Gris-ORF1       AYVYAEIAGYASRGNAFHMTGLRSDGAELAAAIRAALDEARLDASAVDYVNAHGSGTRQN
ElmK            APVLAEVAGFATRANAYHMTGLRSDGREMAAAIDAALLAAGRGPADVDYINAHGSGTRQN
TcmK            AHAYAEVRGFATRSNAFHMTGLKPDGREMAEAITAALDQARRTGDDLHYINAHGSGTRQN
PdmA            AHVYCEVAGYATRGNAYHMTGLKPDGREMAEAIRVAMDAARVAPADLDYINAHGSGTKQN
MtmP            AHVYAEISGFASRCNAYHMTGLRPDGIEMAEAIRTALDEARLDPTAVDYVNAHGSGTKQN
Dau-ORFA        ARIYAEIGGYASRGNAYHMTGLRADGAEMAAAITAALDEARRDPSDVDYVNAHGTATRQN
DpsA            ARIYAEIGGYASRGNAYHMTGLRADGAEMAAAITAALDEARRDPSDVDYVNAHGTATKQN
OxyA            ARAYAEIAGYAGRCNAYSMTGLRSDGRELAEAVSRALDIARVDPSEVDYVNAHGSATKQN
                *     : *:: * .*. ****  .* *:. ::  ::  :      :.*:****:.*:**
```

```
Gra-ORF1         DRHETAAFKRSLGEHAYAVPVSSIKSMGGHSLGAIGSIEIAASVLAIEHNVVPPTANLHT
Qin-ORF19        DRHETAAFKIALGQHAYDVPVSSIKSMVGHSLGAIGSIEIAACALAIKHNVVPPTANLHT
Med-ORF1         DRHETAAFKRSLGDHAYAVPVSSIKSMVGHSLGAIGSIEIAASLLALTHQVVPPTANLHT
ActI-ORF1        DRHETAAYKRALGEHARRTPVSSIKSMVGHSLGAIGSLEIAACVLALEHGVVPPTANLRT
AlnL             DRHETAAFKETLGQHAYEVPISSIKSMVGHSLGAIGSIEIAACALAMENGAVPPTANLHE
FrnL             DRHETAAFKATLGERARSVPVSSIKSMIGHSLGAIGSLEIAAWALAMEYGVVPPTANLDT
AlpA             DRHETAAFKRSLGEHAFRTPVSSIKSMVGHSLGAIGSIEIAASALAMEYDVVPPTANLHT
JadA             DRHETAAFKKSLGDHAYRTPVSSIKSMVGHSLGAIGSIEIAASALAMEHNVVPPTGNLHT
LanA             DRHETAAFKLSLGEHAYRTPISSIKSMVGHSLGAIGSIEIAASLLAMENHVVPPTANLHT
PgaA             DRHETAAFKLSLGDHAYRTPVSSIKSMVGHSLGAIGSIEIAASVLAMKNHVVPPTANLHT
UrdA             DRHETAAFKRSLGDHAYAVPVSSIKSMVGHSLGAIGSIEIAASALAMEHGVVPPTANLHT
SimA1            DRHETAAFKRSLGDHAYRTPVSSIKSMVGHSLGAIGSIEIAACVLAMAESVIPPTANLHD
Aur1D            DLHETAAYKLALGPHAHRTPVSSVKSMIGHSLGSVGSMEIAASVLAMEHHVVPPTANLRT
Gris-ORF1        DRHETVALKSALGQHAHRVPVSAIKSMVGHSLGAIGSIEIAACALAMRHHVIPPTANLHT
ElmK             DRHETAAFKRSFGERAYAIPVSSIKSMIGHSLGAIGSLELAACVLAMESDLIPPTANYAE
TcmK             DRHETAAFKRSLGQRAYDVPVSSIKSMIGHSLGAIGSLELAACALAIEHGVIPPTANYEE
PdmA             DRHETAAFKRSLGERAYELPVSSIKSMVGHSLGAIGSIELAACALAIEHGVVPPTANLHN
MtmP             DRHETAAFKRSLKDHAYRVPVSSIKSMIGHSLGAIGSLEVAASALAIEHDTVPPTANLHD
Dau-ORFA         DRHETSAFKRSLGDHAYRVPISSVKSMIGHSLGAAGSLEVAATALAVEYGAIPPTANLHD
DpsA             DRHETSAFKRSLGEHAYRVPISSIKSMIGHSLGAVGSLEVAATALAVEYGVIPPTANLHD
OxyA             DLHETAAFKRSLGPHAYSVPISSIKSMIGHSLGAICALEVAASALRIEHGVIPPTANLRE
                 * *** * * ::  :*   *:*::*** *****:  ::*:**  * :    :***.*

Gra-ORF1         PDPECDLDYVPLTAREQRVDTVLTVGSGFGGFQSAMVLHRPEEAA----
Qin-ORF19        PDPECDLDYVPLTAREQRVDSVLTVGSGFGGFQSAMILRAPEGVKA---
Med-ORF1         ADPECDLRYVPLTAREAPVRSVLTVGSGFGGFQSAMVLRRPEEAAA---
ActI-ORF1        SDPECDLDYVPLEARERKLRSVLTVGSGFGGFQSAMVLRDAETAGAAA-
AlnL             PDPECDLDYVPNEAREHGVDAVLSVGSGFGGFQSAMVITREETTR----
FrnL             PDPECDLDYVPHEARETRVDRVLSVGSGFGGFQSAMVLTRDTGARLPTA
AlpA             ADPECDLDYVPLVARDQLIDAVLTVGSGFGGFQSAMVLASPERSLV---
JadA             PDPECDLDYVR-SCREQLTDSVLTVGSGFGGFQSAMVLARPERKIA---
LanA             PDPECDLDYVPLVAREHTTDTVLTVGSGFGGFQSAMVLARPERSAA---
PgaA             PDPECDLDYVPLTAREQRTDTVLTVGSGFGGFQSAMVLARPERNAA---
UrdA             PDPECDLDYVPRTARDWKTDAVLSVGSGFGGFQSAIVLARPDRRTA---
SimA1            PEPECDLDYVPITAREARLDRVLSVGSGFGGFQSAMVLTRPERNTA---
Aur1D            PDPECDLDYVPLVARDHRTDAVLTVSSGFGGFQSAMVLARPDRRTA---
Gris-ORF1        PDPECDLDYVPLTARAQRTDAVLTIGSGFGGFQSAMVLARPDRSAA---
ElmK             PDPECDLDYVPNTAREARLDTVVSVGSGFGGFQSAAVLTRPEGRPR---
TcmK             PDPECDLDYVPNVAREQRVDTVLSVGSGFGGFQSAAVLARPKETRS---
PdmA             ADPECDLDYVPLVAREGRIRTVLSVGSGFGGFQSATVLREAA-------
MtmP             RDPACDLDYTPITAREQRTDTVLSVGSGFGGFQSAMVLTAPGLREAA--
Dau-ORFA         PDPELDLDYVPLTAREKRVRHALTVGSGFGGFQSAMLLSRPER------
DpsA             PDPELDLDYVPLTAREKRVRHALTVGSGFGGFQSAMLLSRLER------
OxyA             PDPDCDLDYVPLVAREAEVSTVVSVASGFGGFQSAIVLTEPGRQR----
                  :*  ** *.   .*      .:::.********** ::
```

Figure S19. A comparison of auricin KSα (Aur1D) *from S. lavendulae subsp. lavendulae* CCM 3239 with griseusin KSα (Gris-ORF1) from *S. griseus* K-63 and several representative KSα proteins from main groups of aromatic polyketides. Protein sequences (and accession numbers) are as follows: granaticin Gra-ORF1 (CAA09653), qinimycin Qin-ORF19 (WP_058047374), medermycin Med-ORF1 (BAC79044), actinorhodin ActI-ORF1 (CAC44200), alnumycin AlnL (ACI88861), frenolicin FrnL (AAC18107), kinamycin AlpA (AAR30152), jadomycin JadA (AAB36562), landomycin LanA (AAD13536), gaudimycin PgaA (AAK57525), urdamycin UrdA (CAA60569), simocyclinone SimA1 (AAK06784), auricin Aur1D (AAX57191), griseusin Gris-ORF1 (CAA54860), elloramycin ElmK (CAP12600), tetracenomycin TcmK (AAA67515), pradimicin PdmA (ABM21747), mithramycin MtmP (CAA61989), daunorubicin Dau-ORFA (AAA87618), doxorubicin DpsA (AAA65206), oxytetracycline OxyA (AAZ78325),

```
AlpB            -----MTA-----------------SVVVTGLGVTAPNGLGLKDYWAATLGGKHGIGRIT
JadB            -----MSA-----------------SVVVTGLGVAAPNGLGREDFWASTLGGKSGIGPLT
UrdB            --VNTGAV-----------------EVAVTGLGVVAPNGLGTDAYWAATRKGTSGIARIS
Aur1E           -----MSA-----------------RILVTGIGVAAPSGLGVEDFWSVTRIGKNAIGPVT
Gris-ORF2       -----VSAPGGG----------DRGRTLITGMGLATPHGVDVEDFWAATRVGKNAIGPVT
LanB            -----MTA-----------------RVVITGIGIAAPNGFGVEDYWAATRVGKSAIGRIT
PgaB            -----MST-----------------RTVITGIGVATPNGLGVDEFWAATRVGKNAIARVT
SimA2           -----MTT-----------------SVVVTGLGVAAPNGLGTADYWAATREGRSGIGRVT
ActI-ORF2       -----MS-------------------VLITGVGVVAPNGLGLAPYWSAVLDGRHGLGPVT
Qin-ORF18       -----MTSKDG-----------LNGRTVITGIGVTAPNGLGTEAFWKAVLAGHTGIGPVT
Med-ORF2        -----MS-----------------DRALITGIGVVAPNGLGVKEYWNATLEGRGGIAPLT
Gra-ORF2        -----VSTPD------------RRRAVVTGLSVAAPGGLGTERYWKSLLTGENGIAELS
AlnM            -----MTLATP--AAQETPERTGRPTAVITGIGVAAPNGLGTEQWWQSTLQGTSGIGPVV
FrnM            -----MTTAPSR-TAQGAPPGAALP-PVFTGIGVAAPNGLGTEEWWAATLRGEHGLRPVT
ElmL            VTGSDTEDGST---------------GWITGLGVVAPNGIGAEEYWKATLEGRSGLRTIT
TcmL            -----MSAPAP---------------VVVTGLGIVAPNGTGTEEYWAATLAGKSGIDVIQ
PdmB            -------------------------------VVAPTGIGVEEHWAATLRGVPVIGPLT
MtmK            -----MS--------------ADASQAVITGIGVAAPNGLSVKAWWDAVLRGESGIRRLS
OxyB            -----MTGQLAPAPETGTGRPGGSVRPVVTGLGVVAPNGLGTERYWAATLRGDSGIGRIT
Dau-ORFB        --------------------------VVTGLGIVAPNGLGVGAIWDAVLNGRNGIGPLR
DpsB            MTGTAARTASSQLHASPAGRRGLRGRAVVTGLGIVAPNGLGVGAYWDAVLNGRNGIGPLR
                                                 :.:* * .    *     *   :  :

AlpB            RFDPTGYPARLAGQIDGFEADRLLPSRLLPQTDRVTRL-ALVAADWALADAGADPAQLP-
JadB            RFDPTGYPARLAGEVPGFAAEEHLPSRLLPQTDRMTRL-ALVAADWALADAGVRPEEQD-
UrdB            RFDPSRYPVQLAGEIEGFDAKGHLPGRLLPQTDRMTQL-ALVAADWAFEDAAVRPGDLP-
Aur1E           RFDASAYPSRLAGEIHGFEPKEHLPGRLVPQTDRVTQL-ALVAADCAFADAGIEPGTID-
Gris-ORF2       RFDASGYPARLAGEIRGFSAADHLPGRLVAQTDRVTQL-ALVAADRAFRDAGVAPGDLP-
LanB            RFDPTQYPARLAGEIRGFDARDHLPGRLIPQTDRMTQL-ALVATDSAFEDAGVKPGDIP-
PgaB            HFDPSSYPARLAGEIRGFEAKDHLPSRLIPQTDRMTQL-ALVAADCAFEDAGVELGNIP-
SimA2           RFDPSQYPSRLAGEVPGFVAEDHLPSRLLPQTDHMTRL-ALVSADWALQDAGIRPEELP-
ActI-ORF2       RFDVSRYPATLAGQIDDFHAPDHIPGRLLPQTDPSTRL-ALTAADWALQDAKADPES---
Qin-ORF18       RFDASRYAASLAGQIDDFDAAEHLNSRLLPQTDPSTRL-ALVAADWALTDADVSPDT---
Med-ORF2        RFDASRYPSRLAGQILGFDPAEHLPNRLLPQTDVSTRL-ALVAAEQALADGGVDPAE---
Gra-ORF2        RFDASRYPSRLAGQIDDFEASEHLPSRLLPQTDVSTRY-ALAAADWALADAGVGPES---
AlnM            DYDASRYPSRLVGRIDGFEAAEHIPGRLLPQTDRVTRL-ALVAGAEALADADANPAE---
FrnM            EYDASGHPGGLVGRVPDFDAARHLPGRLLPQTDRVTRL-ALVAADEALKDAAVDPAR---
ElmL            GFDAGQYPVRVAGEVGTFADEPSLSGRILPQTDRMTRY-ALVASDWALADSGVRTDEHDG
TcmL            RFDPHGYPVRVGGEVLAFDAAAHLPGRLLPQTDRMTQH-ALVAAEWALADAGLEPEKQDE
PdmB            RFDASRYPSPFGGEVPGFDAAERVPGRLIPQTDHWTHL-ALAATDLALADAGVVPAELPE
MtmK            RFDPGRYPARLAGEIRDFVDADHVPGRLLPQTDRVTRL-SLAVAREAVEDAGVDLERLP-
OxyB            RFDPSGYTSSLAGEIADF-DPARLPNRLLPQTDLMTRL-ALVAAEEALDDAGADPRTMP-
Dau-ORFB        RFADDGRLGRLAGEVSDFVPEDHLPKRLLVQTDPMTQMTALAAAEWALREAGCAPSS---
DpsB            RFTGDGRLGRLAGEVSDFVPEDHLPKRLLAQTDPMTQY-ALAAAEWALRESGCSPSS---
                 :        . *.:  *     :  *:: ***  *:  :*.    *. :.

AlpB            --------EFDMGVITASAAGGFEFGQGELQALWSQGSQYVSAYQSFAWFYAVNSGQISI
JadB            --------DFDMGVVTASASGGFEFGQGELQKLWSQGSQYVSAYQSFAWFYAVNSGQISI
UrdB            --------EFEMGVITASSSGGFEFGQRELQALWSRGSRYVSAYQSFAWFYAVNSGQISI
Aur1E           --------PYAMGVVTAAGAGGFEFAENELRKLWSEGAKHVSAYQSFAWFYAVNSGQISI
Gris-ORF2       --------ANGMGVVTAAGSGGFEFGERELRKLWSLGANHVSAYQSFAWFPTANTGQIAI
LanB            --------EYDMGVVTASTAGGFEFGQNELQALWSKGSQHVSAYQSFAWFYAVNSGQISI
PgaB            --------AYDMGVVTASTSGGFEFGQNELKKLWSQGSRYVSAYQSFAWFYAVNSGQISI
SimA2           --------EYAAGVVTASSAGGFEFGQNELRALWSKGSQYVSAYQSFAWFYAVNTGQISI
ActI-ORF2       -LT-----DYDMGVVTANACGGFDFTHREFRKLWSEGPKSVSVYESFAWFYAVNTGQISI
Qin-ORF18       -LP-----DYDMGVVTSNALGGFDFTHREFDKLWNKGPDFVSVYESFAWFYAVNTGQISI
Med-ORF2        -LV-----DFDLGVITSNASGGFAFTHREFANLWSKGPEYVSVYESFAWFYAVNTGQVSI
Gra-ORF2        GLD-----DYDLGVVTSTAQGGFDFTHREFHKLWSQGPAYVSVYESFAWFYAVNTGQISI
AlnM            LAEQDGYGEYGCGVVTSNATGGFEFTHREIRKLWTQGPQQVSVYESFAWFYAVNTGQLSI
FrnM            LP------EYGASAVTSNATGGFEFTHREIRKLWTEGPARVSVYESFAWFYAVNTGQISI
ElmL            ---------FSTGVITASAAGGFEFGQRELQKLWGSGPGEVSAYQSFAWFYAVNTGQISI
TcmL            ---------YGLGVLTAAGAGGFEFGQREMQKLWGTGPERVSAYQSFAWFYAVNTGQISI
PdmB            ---------YEMAVVTASSSGGVEFGQREIQALWRDGPRHVGAYQSIAWFYAATTGQISI
MtmK            --------RYAAGVSTASSAGGFEFGQRELQALWSKGGQYVSAYQSFAWFYAVNTGQISI
OxyB            --------DFAAGVVTAASAGGFDFGQRELEALWSKGGAHVSAYQSFAWFYPVNSGQISI
Dau-ORFB        --------PLEAGVITASASGGFASGQRELQNLWSKGPAHVSAYMSFAWFYAVNTGQIAI
DpsB            --------PLEAGVITASASGGFAFGQRELQNLWSKGPAHVSAYMSFAWFYAVNTGQIAI
                            .. *:   **.   . *:  **  *   *..* *:***  ...:**:*
```

```
AlpB            RNGMKGPAGVVVSEGAGGLDAVAQARRQIRKG-TSLIVTGAVDASLCPWGWVAQLAGGRL
JadB            RNGMKGPSGVVVSDQAGGLDAVAQARRQIRKG-TRLIVSGGVDASLCPWGWVAHVASDRL
UrdB            RNGMRGPSGVVVSDQAGGLDAVAQARRQIRKG-TRLVMSGAVDASICPWGWVAQMASNRL
Aur1E           RNGLRGPAGVVISDQAGGLDALAQARRQLRKG-SKLIATGGFDAPICSLGWASHLHGGLM
Gris-ORF2       RSDSRGPNGVVVGDQAGGLDALGQARRLIRRG-TGLVAAGGFEASLTPLGWTARLSTGLM
LanB            RNGMRGHSAVVVSDQAGGLDAIAQARRQIRKG-SKLICSGGVDASICPWGWVAQLANGRV
PgaB            RNGMKGPSGVVVSDHAGGLDAIAQARRQIRKG-SKLIFSGGFDASICPWGWAAQIAGGRL
SimA2           RHGLRGPSGVVVSDQAGGLDALAQARRQIRRG-SQVIVSGGIDASICSWGWGAQLTSGRM
ActI-ORF2       RHGMRGPSSALVAEQAGGLDALGHARRTIRRG-TPLVVSGGVDSALDPWGWVSQIASGRI
Qin-ORF18       RHKLRGPSAALVGEQAGGLDALGHARRTIRRG-TPLVVAGGVDSALDPWGYVSQLAGGRV
Med-ORF2        RHNVRGPGAALVAEQAGGLDALGHARRSLRLG-TPLVVTGGVDSALDPWGWASHLSSGLI
Gra-ORF2        RNTMRGPSAALVGEQAGGLDAIGHARRTVRRG-PGWCSAVASTRRSTRGASSSQLSGGLV
AlnM            RHKLRGPSGVLVSEQAGGLDAIGQSRRTLRQG-VKLSLTGGMDSSLDPWGLVSHLASGRL
FrnM            RHGMRGPGAVVVADQAGGLDALGQARRVLRKG-GVLAVSGGVESALDPWGLAAHASSGTL
ElmL            RHGLRGHSSVAVAEQAGGLDAVAQATRLVDHGTLRVAVAGGFEAPVSPWGLVAQIPSGLL
TcmL            RHGMRGHSSVFVTEQAGGLDAAAHAARLLRKGTLNTALTGGCEASLCPWGLVAQIPSGFL
PdmB            RHGMRGPCGVVVAEQAGALESFAQARRYLADG-ARVVVSGGTDAPFSPYGLTCQLGSGRL
MtmK            GYDLRGPSGVLVTEQAGGLDAVAQARRLLRRG-SELMVTGGVDSALCPWGWTAHLAGGRL
OxyB            RHDMRGPGGALVAEQAGGLDAVAKARRHVRDG-TPLMLTGGVDGSLCPWGWLCMTRSGAL
Dau-ORFB        RHDLRGPVGVVVAEQAGGLDALAHARRKVRGG-AELIVSGAMDSSLCPYGMAAQVRSGRL
DpsB            RHDLRGPVGVVVAEQAGGLDALAHARRKVRGG-AELIVSGAVDSSLCPYGMAAQVKSGRL
                    :*  .. : : **.*:: .:: * :  *      : .        .  .    . :

AlpB            STSDEPDHAYLPFDRDARGFVPGEGGAILIAEDAAAARTRGAR-PYGEIAGYGATIDPRP
JadB            STSEEPARGYLPFDREAQGHVPGEGGAILVMEAAEAARERGAR-IYGEIAGYGSTFDPRP
UrdB            STSRDPERAYLPFDDAAGGHVPGEGGALLVLEELEQARARGARQIYGVIAGYGSTLDPRP
Aur1E           STSDEPERAYLPFDDAAAGYVPGEGGAMLILEDEDSARDRGARTVYGEFAGYGATLDPKP
Gris-ORF2       STSDDPDRAYLPFHPAARGYVPGEGGALLILEDERSALARGAATAHAELAGYSATLDPRP
LanB            STSENTERAYLPFDADASGYVPGEGGALLILEDAEAARQRGAENVYGEIAGYGSTFDPRP
PgaB            SHSDRPERAYLPFDRAANGYVPGEGGALLILEDERTARERGAEKIYGEVAGYGSTFDPRP
SimA2           STDDDPATAYRPFDGAAAGHVPGEGGALLVLEEAGAAAARGAR-VYGRIAGYGTTFDPKP
ActI-ORF2       STATDPDRAYLPFDERAAGYVPGEGGAILVLEDSAAAEARGRHDAYGELAGCASTFDPAP
Qin-ORF18       TTATDPARAYLPFSADASGYVPGEGGAILVAEDEASARERGVQQVYGELAGYAATFDPAP
Med-ORF2        SDSDDPDRAYLPFDARARGHVPGEGGAFLVMEDEQGALRRGAGQVYGELAGYAATFDPHP
Gra-ORF2        STVADPERAYLPFDVDASGYVPGEGGAVLIVEDADSARARGAERIY---VRSPLRRDPAP
AlnM            SRSDDPATAYLPFDTRAAGQVPGEGGAMLVLEEETAARARGA-RVYGEIAGYAATFSPRP
FrnM            SRSGDPATAYLPFDRRALGTVVGEGGALLTLETPRHAEERDAPRIYGELAGYAATFDPPA
ElmL            STVADPARAYLPFDPDASGWVPGEGGAALVVERAADARARGADHVYGRIAGHASTFDPRP
TcmL            SEATDPHDAYLPFDARAAGYVPGEGGAMLVAERADSARERDAATVYGRIAGHASTFDARP
PdmB            STGADPARAYLPFDAAANGFVPGEGGAILIIEQAATAQDR----SYGRIAGYAATFDPPP
MtmK            STGDDPARAYLPFSADADGEVVGEGGALLVLERAAAAPSRGA-RVYGVFAGYAATFDPPP
OxyB            TTRTDPRRAYLPFSPDASGYVPGEGGALLVLEDPRAAAERGAPQVYGRIAGYAATFDPRP
Dau-ORFB        SGSDDPTAGYLPFDRRAAGHVPGEGGAILAVEDAERVAERGGK-VYGSIAGT-ASFDPPP
DpsB            SGSDNPTAGYLPFDRRAAGHVPGEGGAILTVEDAERAAERGAK-VYGSIAGYGASFDPPP
                :    .  .* **   * * * ***** *  *    .  *     :   .      .. .

AlpB            GSGREPGLRKAIETALADARLSAADIDVVFADGAGDPAGDRIEADAISTVFGDRGVPVTV
JadB            GSGREPGLRKAIELALADAGAAPGDIDVVFADAAAVPELDRVEAEALNAVFGTGAVPVTA
UrdB            GSGRPAGLRKAIELALADAGAAPEEIDVVFADAAAVPELDRIEAEALTQVFGARAVPVTA
Aur1E           GSGREPGLRRAIDVALTDAACHPAEVDVVFADGAATPRLDREEAEAITAVFGPRAVPVTV
Gris-ORF2       GSGREPGLRRAIELALADAGVAPAEVDVVFADAAGSPEPDAVEVAAVAGVFGPRAVPVTA
LanB            GSGREPGLRRAIELALADADAEPSDIGVVFADAAGTPELDRVEAEALIEVFGAAGVPVTA
PgaB            GSDREPGLRHAIEVALADAGVTAADVDVVFADAAGSPDLDRQEADAITAVFGPSGVPVTA
SimA2           GSGREPGLRRAIELALTDAGLAPADIDVVFADAAAVPDLDRIEARALVEVFGPRGLPVTA
ActI-ORF2       GSGRPAGLERAIRLALNDAGTGPEDVDVVFADGAGVPELDAAEARAIGRVFGREGVPVTV
Qin-ORF18       GTGRPPALRRAAEAALADAGLAPGDIDVVFADGAGVPELDAAEAAAISGLFGAGAVPVTA
Med-ORF2        DSGRPPALRRAAELAIADAGLEPSDIGVVFADAAGSADLDRTEAEAIAAVFGPRGVPVAA
Gra-ORF2        GSGRPPALGRAAELALAEAGLTPADISVVFADGAGVPELDRAEADTLARLFGPRGVPVTA
AlnM            GSGRPPGLERAARLALADSGLGVEDVDVVFADAAGLPTADDEEAAALRALFGPYGVPVTA
FrnM            GSGRPPGLERAARLALADAGLAPGDVDVVFADAAGLPAADAEAAALRALFGPGGVPVSV
ElmL            GLGRPPALARAVRTALDRARTHPGDIDVVFADAAGLPEQDAAEVAALTEVFGSRKVPVTA
TcmL            GTGRPTGPARAIRLALEEARVAPEDVDVVYADAAGVPALDRAEAEALAEVFGPGAVPVTA
PdmB            GSGRPPTLERAVRAALDDARLTPADVDVVFADAAGVPDLDRAEADAIGAVFGPRGVPVTA
MtmK            GRGGTPQLARAAELALVDAGMTPDQVDVVFADAAGERAADRAEADALTRVFGARGVPVTA
OxyB            GSGREPGLRRALRLALDDAGIGPADVDVVFADAMGVPALDAVETQVLAAEFGPRGVPVTA
Dau-ORFB        GSGRPSALARAVETALADAGLDRSDIAVVFADGAAVGELDVAEAEALASVFGPHRVPVTV
DpsB            GSGRPSALARAVETALADAGLDGSDIAVVFADGAAVPELDAAEAEALASVFGPRRVPVTV
                . .  .   :*   *:  :     :: **:**. .    *  *. .:   **   :**:.
```

```
AlpB            PKTMTGRLYSGGAPLDLAAAFLALRDGVIPPTVHID-PCADYPLDLVLGEPR-PAPLRTA
JadB            PKTMTGRLYSGAAPLDLAAAFLAMDEGVIPPTVNVE-PDAAYGLDLVVGGPR-TAEVNTA
UrdB            PKTMTGRLYSGAAPLDLAAAFLAMRDGVVPPSVGVS-PSPDHDLDLVVHQER-AMTVRSA
Aur1E           PKTMTGRINSGGAPIDVVSAVLSMREGLIPPTTNVE-LSDAYDLDLVAVRPR-TASVRTA
Gris-ORF2       PKTMIGRLQAGGAPVDVVTAVLAIREGLIPPTADAE-SATARELDLVVGRPR-TASVGTA
LanB            PKTMTGRLYSGAAPVDVVTAVLAMREGLIPPTVNVS-LSPEYDIDLVTAQPR-TARVRNA
PgaB            PKTMIGRLYSGAAPVDVVAAVLAIREGLIPPTTNVE-LSPDYDLDLVTGQPR-TASVRTA
SimA2           PKSMTGRLNSGAGSLDVATALLAIRDGVIPPTINVT-AQDDYELDLVTAQAR-SARLRSA
ActI-ORF2       PKTTTGRLYSGGGPLDVVTALMSLREGVIAPTAGVTSVPREYGIDLVLGEPR-STAPRTA
Qin-ORF18       PKALTGRLYSGGGPLDVATALLSIRDGVIPPTPTGAPVPEDYGLDLVQGAPR-DQAVRTA
Med-ORF2        PKALTGRLLAGGGPLDVVAASVRLRDGLLPAVPYEGETPDAYGIDLVRGTPR-PTSARAA
Gra-ORF2        PKALTGRLCAGGGPADLAAALLALRDQVIPATGRHRAVPDAYALDLVTGRPR-EAALSAA
AlnM            PKTLTGRLFGGGAPLDVAAALLALRDGVIPPTAGIDRPVPEHRLDLVRGTPR-HTPLRTA
FrnM            PKTQTGRLASGGPALDVAAALLALRDGLVPPAVHLDEVDPAYGLDLVRDTPR-ALPLRTA
ElmL            PKTMTGRLYSGGGALDTATALLALRDGVVPPTVGTRTAPP--ELDLVLGAPR-DLPLRNA
TcmL            PKTMTGRLYAGGAALDVATALLSIRDCVVPPTVGTGAPAPGLGIDLVLHQPR-ELRVDTA
PdmB            PKSLTGRLYAGGPALDAATALLAMHDSVIPPTAGGADVPPGYALDLVGAEPR-PARLRTA
MtmK            PKTMTGRLSAGGASLDLAAALLALRDQVVPPTVNVTEPADDCPVDLVTGRPR-PLPLRAA
OxyB            PKTMTGRLLAGGASLDLAAALLSLRDQVVPPTVHVDGGEIPDSLDLVTGAPR-PARLRHA
Dau-ORFB        PKTLTGRLYSGAGPLDVATGLLALRDEVVPATGHVH-PDPDLPLDLVVTGRPRAMADARAA
DpsB            PKTLTGRLYSGAGPLDVATALLALRDEVVPATAHVD-PDPDLPLDLVVTGRPRSLADARAA
                **:  **: .*. . * .:. : : : ::..            :*:*    *       *

AlpB            LILARGHGGFNSAMAVRAV--------
JadB            LVIARGHGGFNSAMVVRSAN-------
UrdB            LVIARGHGGFNSAVVVRAVG-------
Aur1E           LVLARGRGGFNSAVVVRAVD-------
Gris-ORF2       LVLARGHGGFNSAVVLRAVD-------
LanB            LVVARGYGGFNSAMVVRGTDR------
PgaB            LVLARGVGGFNSAVVVRAVD-------
SimA2           LVVARGHGGFNSALVVQDAA-------
ActI-ORF2       LVLARGRWGFNSAAVLRRFAPTP----
Qin-ORF18       LVLARGRHGFNSAVVVRA---------
Med-ORF2        LVLARGRWGFNSAVVVKAADRG-----
Gra-ORF2        LVLARGRHGFNSAVVVTLRGSDHRRPT
AlnM            LVLARGHGGFNAAVVVR---APEAA--
FrnM            LVLARGHGGFNAAVVVRGRRRPRTA--
ElmL            LVLARGTGGFNSAVVVARV--------
TcmL            LVVARGMGGFNSALVVRRHG-------
PdmB            LIIARGYGGFNAALVLRGPNT------
MtmK            LVLARGRGGFNAAAVVRALS-------
OxyB            LVLARGHGGFNSAMVVSGRD-------
Dau-ORFB        LVVARGHGGFNSALVVRGAA-------
DpsB            LLVARGYGGFNSALVVRGAA-------
                *::***  ***:* .:
```

Figure S20. A comparison of auricin KSβ (Aur1E) *from S. lavendulae subsp. lavendulae* CCM 3239 with the griseusin KSβ (Gris-ORF2) from *S. griseus* K-63 and several representative KSβ proteins from main groups of aromatic polyketides. Protein sequences (and accession numbers) are as follows: kinamycin AlpB (AAR30151), jadomycin JadB (AAB36563), urdamycin UrdB (CAA60570), auricin Aur1E (AAX57192), griseusin Gris-ORF2 (CAA54859), landomycin LanB (AAD13537), gaudimycin PgaB (AAK57526), simocyclinone SimA2 (AAK06785), actinorhodin ActI-ORF2 (CAC44201),qinimycin Qin-ORF18 (WP_058047373), medermycin Med-ORF2 (BAC79045), granaticin Gra-ORF2 (CAA09654), alnumycin AlnM (ACI88862), frenolicin FrnM (AAC18108), elloramycin ElmL (CAP12601), tetracenomycin TcmL (AAA67516), pradimicin PdmB (ABM21748), mithramycin MtmK (CAA61990), oxytetracycline OxyB (AAZ78326), daunorubicin Dau-ORFB (AAA87619), doxorubicin DpsB (AAA65207).

```
Gris-ORFX        ---IQNSRNDPRFVQISHELKPFIEPYDPETWRTPADAMATRFYDWSAGE
Aur1C            GQRIQESRTDPRFMRISHDLLPFIQPYDPATWRTPADAMATRFYDWSASE
                    **:**.****::***:* ***:**** ******************.*
```

Figure S21. A comparison of auricin CYC Aur1C (AAX57190) *from S. lavendulae subsp. lavendulae* CCM 3239 with the griseusin partial CYC (Gris-ORFX) from *S. griseus* K-63.

```
Gris-ORFY        -VPEDDDARIVVVGAGPVGLLLAGLLRRSGADVVVLEQLTAPTTESRASTLHARTMEILD
Aur1I            MSGDYSETQVIVVGAGPVGLLLAGELRLAGTDVVVLDKLTAPTTESRASTLHSRTMEILD
                    : .:::::************* ** :*:*****::**************:*******

Gris-ORFY        GCGVRDRLGAVEREPRGHFGGIGLDLTLPVPRPGQWKVPQTRLEEVLARWARELGVRI--
Aur1I            TRGLLDGLGDIPNDPSGHFGGIPFDLALPGPYPGQWKVAQTRLEEVLGQWAADLGADIRR
                   *: * ** : .:* ****** :**:** * ******.********.:** :**. *
```

Figure S22. A comparison of auricin oxygenase Aur1I (AAX57196) *from S. lavendulae subsp. lavendulae* CCM 3239 with the griseusin partial oxygenase (Gris-ORFY) from *S. griseus* K-63.
